# Neuronal pSTAT1 hallmarks synaptic pathology in autoimmune encephalitis against intracellular antigens

**DOI:** 10.1007/s00401-025-02882-7

**Published:** 2025-04-25

**Authors:** Giovanni Di Liberto, Kristof Egervari, Alberto Vogrig, Marianna Spatola, Margot Piccinno, Ilena Vincenti, Ingrid Wagner, Mario Kreutzfeldt, Verena Endmayr, Karoline Ostertag, Jasmin Rahimi, Alex Vicino, Anne-Katrin Pröbstel, David Meyronet, Stephan Frank, Marco Prinz, Ekkehard Hewer, Jean-Philippe Brouland, Laurence de Leval, Laura Parkkinen, Bogdan Draganski, Virginie Desestret, Divyanshu Dubey, Sean J. Pittock, Shanu F. Roemer, Dennis W. Dickson, Romana Höftberger, Sarosh R. Irani, Jérôme Honnorat, Renaud Du Pasquier, Doron Merkler

**Affiliations:** 1 Department of Pathology and Immunology, University of Geneva, Geneva, Switzerland; 2 Department of Clinical Neurosciences, Neurology Service, Lausanne University Hospital and University of Lausanne, Lausanne, Switzerland; 3 Division of Clinical Pathology, Geneva University Hospital, Geneva, Switzerland; 4 Department of Medicine (DMED), University of Udine, Udine, Italy; 5 Clinical Neurology, Department of Head-Neck and Neuroscience, Azienda Sanitaria Universitaria Friuli Centrale (ASU FC), Udine, Italy; 6 Neuroimmunology Program, August Pi i Sunyer Biomedical Research Institute (IDIBAPS), Hospital Clinic, University of Barcelona and Caixa Research Institute (CRI), Barcelona, Spain; 7 Division of Neuropathology and Neurochemistry, Department of Neurology, Medical University of Vienna, Waehringer Guertel 18-20, 1090 Vienna, Austria; 8 Comprehensive Center for Clinical Neurosciences and Mental Health, Medical University of Vienna, Waehringer Guertel 18-20, 1090 Vienna, Austria; 9 Department of Neurology, Clinic Hietzing, Vienna, Austria; 10 Department of Neurology and Karl Landsteiner Institute for Neuroimmunological and Neurodegenerative Disorders Klinik Donaustadt, Vienna, Austria; 11 Department of Neurology and Research Center for Clinical Neuroimmunology and Neuroscience Basel (RC2NB), University Hospital Basel and University of Basel, Basel, Switzerland; 12 Departments of Biomedicine and Clinical Research, University Hospital Basel and University of Basel, Basel, Switzerland; 13 Center of Neurology, Department of Neuroimmunology, University Hospital and University Bonn, Bonn, Germany; 14 German Center for Neurodegenerative Diseases (DZNE), Bonn, Germany; 15 Institute of Neuropathology, Hospices Civils de Lyon, 69008 Lyon, France; 16 Centre de Recherche en Cancérologie de Lyon, Inserm U1052, CNRS UMR5286, CEDEX 08, 69373 Lyon, France; 17 Université de Lyon, Université Claude Bernard Lyon 1, CEDEX 08, 69373 Lyon, France; 18 Department of Neuropathology, Institute of Pathology, Basel University Hospital, Basel, Switzerland; 19 Institute of Neuropathology, University of Freiburg, Freiburg, Germany; 20 Signaling Research Centers BIOSS and CIBSS, University of Freiburg, Freiburg, Germany; 21 Department of Laboratory Medicine and Pathology, Institute of Pathology, Lausanne University Hospital and University of Lausanne, Lausanne, Switzerland; 22 Nuffield Department of Clinical Neurosciences, University of Oxford, Oxford, UK; 23 Universitätsklinik für Neurologie, Inselspital, University of Bern, Bern, Switzerland; 24 University Institute for Diagnostic and Interventional Neuroradiology, Inselspital, University of Bern, Bern, Switzerland; 25 French Reference Center On Paraneoplastic Neurological Syndromes and Autoimmune Encephalitis, Hospices Civils de Lyon, MeLiS – UCBL – CNRS UMR 5284 – INSERM U1314, Université de Lyon, Université Claude Bernard Lyon 1, Lyon, France; 26 Department of Laboratory Medicine and Pathology, Mayo Clinic College of Medicine, Rochester, MN USA; 27 Center for Multiple Sclerosis and Autoimmune Neurology, Mayo Clinic College of Medicine, Rochester, MN USA; 28 Department of Neuroscience, Mayo Clinic, Jacksonville, FL 32224 USA; 29 Department of Neurology, Mayo Clinic, Jacksonville, FL 32224 USA; 30 Autoimmune Neurology Group, West Wing, Level 3, John Radcliffe Hospital, University of Oxford, Oxford, UK

**Keywords:** Phagocytes, Neuroinflammation, Neurodegeneration, Synapses, Resident memory T cells

## Abstract

Autoimmune encephalitis (AE) is an inflammatory syndrome of the central nervous system (CNS) triggered by aberrant immune responses against neuronal intracellular (IC-AE) or surface (NS-AE) autoantigens. The resulting neuronal alterations and clinical trajectories differ, with IC-AE often leading to fatal outcomes. Unfortunately, the scarce availability of tissue from AE cases has hampered systematic analyses that would allow an understanding of the pathogenesis underlying neuronal alterations in T cell-mediated AE syndromes. Here, we assembled a cohort comprising both NS-AE (n = 8) and IC-AE (n = 12) from multiple institutions to delineate key histopathological features that distinguish neuronal pathology between IC-AE and NS-AE. In contrast to NS-AE, IC-AE lesions present a prominent neuronal pSTAT1 signature, accompanied by a high proportion of brain-resident memory CD8 + T cells and neurodegenerative GPNMB + phagocytes which show synaptic engulfment with little C3-complement deposition. Our findings highlight distinct histopathological features of IC-AE compared to NS-AE, providing actionable biomarkers for diagnostics and treatment strategies.

**Supplementary Information:**

The online version contains supplementary material available at 10.1007/s00401-025-02882-7.

## Introduction

Autoimmune encephalitis (AE) represents a heterogeneous spectrum of neuroinflammatory conditions stemming from an aberrant immune response targeting autoantigens within the central nervous system (CNS). The expanding frontiers of this field are driven by the identification of a myriad of associated autoantibodies defining recognizable syndromes with distinct cognitive, epileptic, and movement disorder phenotypes [36, 39, 47].

These autoantibodies exhibit a propensity to target either neuronal surface antigens (NS-AE) or intracellular antigens (IC-AE) [12]. This dichotomous classification holds crucial clinical significance, as it delineates distinct pathogenic drivers and prognosis among AE patients.

Notably, most NS-AE patients are thought to have pathogenic autoantibodies, and these syndromes typically associate with a favorable prognosis and a low rate of paraneoplastic cancers [7, 51]. In contrast, IC-AE patients show a poorer prognosis, often associated with a cancer, with fewer than 10% achieving substantial or full recovery. In IC-AE, a predominant pathogenic role is ascribed to CD8 + T cells [6].

Conversely, the enigmatic cases of antibody-negative AE (AN-AE) demonstrate an intermediate prognosis, with approximately half of these patients exhibiting a favorable outcome [28]. This characteristic suggests AN-AE may include cases from both NS-AE and IC-AE groups, despite target antigens remaining elusive.

Another group appear to have an infectious trigger to their NS-AE, whereby AE follows Herpes Simplex Virus (HSV) encephalitis by several weeks. Notably, the neurological prognosis in these post-infectious cases tends to be more severe compared to those with the typical form of AE [3]. This increased severity may be due to the initial damage from the viral encephalitis along with additional pathomechanisms that remain unclear, which have received limited exploration.

Understanding the cellular and molecular mechanisms underlying neuronal damage in IC-AE is a key step towards improving diagnosis and choosing effective treatment approaches [43].

CD8 + T cells are known to dominate the histological picture of IC-AE and participate in neuronal damage via Granzyme B (GZMB) release [6], however only recently their tissue-resident memory (T_RM_) phenotype has been described in a few cases of paraneoplastic AE [18]. Separately, these T_RM_ cells have been described in infectious diseases, as a population of long-lived memory T cells residing in barrier tissues after the resolution of the initial infection [33]. As a reservoir of memory T cells within these tissues, they can respond to threats or drive immunopathology independently of T cells circulating in the bloodstream [33]. However, evidence of a putative detrimental role for T_RM_ cells for inflammatory diseases is only beginning to emerge, and their presence has been documented in tissues other than barrier tissues, especially during another form of CNS autoimmune disease [54], multiple sclerosis [16, 30].

T_RM_ cells express CD69 and CD103, which are indicative of their resident and persistent phenotype, and features of homeostatic proliferation and survival, such as Bcl-2, along with effector function, such as Granzyme B and Interferon-gamma (IFN-γ) production [33, 42, 45, 55]. The compartmentalized response of T_RM_ cells during CNS autoimmunity appears to be refractory to standard treatments targeting the circulating pool of CD8 + T cells [54]. Therefore, elucidating the role of T_RM_ cells in promoting chronic autoimmune CNS tissue damage represents an important challenge given their long-lasting inflammatory potential.

We have previously shown that neuronal exposure to IFN-γ leads to the phosphorylation and nuclear translocation of pSTAT1 in a murine model of T cell-mediated encephalitis [26]. In both mouse model and human viral encephalitis, as well as human forms of AE, like Rasmussen’s encephalitis and some cases of GAD-antibody encephalitis, signaling through neuronal pSTAT1 appeared critical for driving the acute phagocytic elimination of synapses following CD8^+^ T cell-mediated attack [14]. Subsequent investigations have highlighted the expression of neuronal pSTAT1 in cases of Yo-antibody and Ma2-antibody paraneoplastic cerebellar degeneration (PCD) [59, 62], temporal lobe epilepsy associated with GAD-antibodies [49] and progressive encephalopathy with rigidity and myoclonus (PERM) [60].

Synaptic pathology is generally considered as one of the earliest hallmarks of cognitive impairment in numerous neurodegenerative conditions [58]. In AE, distinctive psychopathology and seizures, in addition to short-term memory loss represent cardinal features [1, 22, 40, 48] and synaptic dysfunction likely underlies these symptoms. Multiple mechanisms may be responsible for autoimmune synaptopathies, including the disruption of protein–protein interactions or internalization of receptors mediated by autoantibodies [11]. However, it is still unclear whether these or other mechanisms might be responsible for the cognitive deficits persisting beyond functional neurological recovery [23].

CNS phagocytes are crucial in modulating synaptic function in health and disease [38] and can acquire reactive states during neurodegeneration characterized by the expression of multiple markers such as GPNMB, SPP1, LGALS3, CTSB [8, 13]. Microglia-mediated synapse engulfment has been shown to involve the classical complement cascade [46]. Specifically, the complement protein C3 has been found to tag synapses for elimination via C3 receptor (CR3)-mediated phagocytosis [41] during visual system development [46] but also viral encephalitis [53], Alzheimer’s disease [24] and demyelinating diseases [57]. The role of complement deposition in AE [25, 32, 63] and the role of CNS phagocytes in this group of conditions remains largely unknown. Additionally, C3 complement-independent mechanisms relying on JAK2-STAT1 signaling pathways are also described [14, 61].

Previous investigations on AE have provided some insights into the pathogenesis of the disease and underscored the involvement of the pSTAT1 pathway in isolated cases; however, the scarcity of histological tissue samples has precluded a broader analysis spanning different IC-AE and NS-AE types, which would be needed to refine disease classifications and inform clinical treatment strategies.

Here, we have assembled an extensive cohort by collecting brain samples from multiple centers, enabling a more systematic comparison across a broader spectrum of AE cases.

Our findings show that neuronal pSTAT1 signaling and abundant brain-infiltrating CD8 + T cells adopting a tissue-resident memory-like phenotype (T_RM_) represent distinct features in IC-AE and post-infectious AE, in contrast to NS-AE. In addition, in IC-AE and post-infectious NMDAR-antibody AE, GPNMB + phagocytes engage in synaptic engulfment without detectable C3 deposition. Our findings suggest that neuronal pSTAT1 signature serves as a potential valuable surrogate biomarker for T cell-driven encephalitis directed against neurons. Furthermore, interfering with this pathway may serve as a viable therapeutic target for encephalitis against intracellular antigens.

## Materials and methods

### Human samples

All tissue samples were examined by at least two independent board-certified neuropathologists who confirmed the diagnosis. Brain biopsies or autopsies from patients with autoimmune encephalitis with antibodies against neuronal surface antigens (NS-AE, n = 8, 5 males and 3 females, [53.8 ± 22.7 years, mean ± SD]), patients with autoimmune encephalitis (or sensory neuronopathy) with antibodies against intracellular antigens (IC-AE, n = 12, 4 males and 8 females, [57.9 ± 20.0 years, mean ± SD]) and non-neurological disease (NND) controls n = 14 (5 male and 9 females, [58.1 ± 20.3 years) were obtained from the collection of the Department of Neuropathology of the Lausanne University Hospital (CHUV, CH), the French Reference Center on Paraneoplastic Neurological Syndromes and Autoimmune Encephalitis and Hospices Civils de Lyon Biological Resource Center, Brain Bank—Tissu Tumorothèque Est (Hospices Civils de Lyon, FR), the Oxford Brain Bank (UK), the Mayo Clinic Jacksonville (USA), the Division of Neuropathology and Neurochemistry of the Department of Neurology of Vienna (AT), the Department of Neuropathology of the University of Basel (CH), the Institute of Neuropathology of the University of Freiburg (DE), and the Department of Neuropathology of Geneva University Hospitals (HUG, CH), IDIBAPS Hospital Clinic and University of Barcelona (ES). Their use for scientific purposes was in accordance with institutional ethical guidelines and was approved by the local ethics committee. Inclusion criteria were: (1) positive autoantibodies in CSF or serum and (2) sufficient archival tissue for pathological analysis. The analyzed sections primarily originated from clinically symptomatic regions, radiologically identified abnormalities (e.g., biopsy sites), or areas with neuroinflammatory changes (T cells or CD68 expression) in routine staining when other data were unavailable. For each patient, approximately 10–15 slices from 1–2 blocks were studied.

Within samples of a study group (NS-AE, IC-AE, NND), no statistical differences between sex were observed regarding the obtained results. Available clinical information is indicated in Table S1 and Table S2. AE cases were matched for brain region, sex, and age as indicated in Figure S1a.

### Comparative pathway analysis of T cell-mediated encephalitis using murine and human datasets

To explore common molecular pathways in T cell-mediated encephalitis, we conducted a comparative analysis using both murine and human datasets. We selected the top 400 upregulated transcripts from our previous work on the neuronal translatome of the “Viral Déjà Vu” murine model of T cell-mediated encephalitis (GSE110593) [14] and the top 400 upregulated proteins in the cerebrospinal fluid (CSF) of patients diagnosed with autoimmune encephalitis against intracellular antigens, utilizing the SomaScan proteomic platform [35]. A Venn diagram was employed to visualize the overlap, identifying shared biomarkers. For pathway enrichment analysis, we used Enrichr [27], selecting the “MSigDB Hallmark 2020” gene set to pinpoint key biological processes and pathways. The expression profiles of these shared biomarkers were further illustrated in a heatmap, categorized by encephalitis subtype and CSF protein expression levels based on the human dataset [35].

### Histology

CNS tissue was fixed with 4% paraformaldehyde (PFA) and was embedded in paraffin as described previously. For immunofluorescence staining, after antigen retrieval (Sodium Citrate pH6, 30 min) and blocking of unspecific binding (fetal calf serum 10% in PBS), PFA-fixed sections were incubated with primary antibodies. Bound antibodies were visualized with appropriate species-specific Cy2-, Cy3-, or Cy5-conjugated secondary antibodies or anti–rabbit tyramide signal amplification (TSA). Nuclei were stained with DAPI (Invitrogen). Immunostained sections were scanned using Pannoramic 250 FLASH II (3DHISTECH) Digital Slide Scanner with objective magnification of 20 × or 40x. Positive signals were quantified by a blinded experimenter using Pannoramic Viewer software (3DHISTECH) and ImageJ/FIJI (NIH Image analysis) and an image analysis ruleset based on Visiopharm. For representative images, white balance was adjusted, and contrast was linearly enhanced using the tools levels, curves, brightness, and contrast in Adobe Photoshop CC. Image processing was applied uniformly across all images within a given dataset.

### Antibodies

Primary antibodies: rabbit anti-IBA1 (polyclonal, 1:100, WAKO catalogue number 019-19741, directly labelled with anti-rabbit AlexaFluor 647), rabbit anti-P2RY12 (polyclonal, 1:200, Sigma-Aldrich catalogue number HPA014518), goat anti-GPNMB (polyclonal, 1:200, RD Systems, catalogue number AF2550), mouse IgG3 anti-CD68 (clone PG-M1, 1:100, DAKO, catalogue number M0876), mouse IgG2b-anti-CD8 (clone 4B11, 1:40, Thermo Fisher Scientific catalogue number MA1-80,231), rabbit anti-NeuN (clone EPR12763, 1:50, Abcam catalogue number ab190195, directly labelled with AlexaFluor 488), mouse IgG2b anti-HuC/D (clone 16A11, 1:100, Invitrogen catalogue number A-21271), rabbit anti-pSTAT1 (clone 58D6, 1:1000, Cell Signalling Technology catalogue number 9167), mouse IgG1 anti-synaptophysin (clone 27G12,1:50, Leica catalogue number NCL-L-SYNAP-299), rabbit-anti-CD69 (polyclonal, 1:50, Sigma-Aldrich catalogue number HPA050525), mouse IgG2a anti-GZMB (clone GrB-7, 1:20, Monosan catalogue number 7029), mouse IgG1-anti-BCL2 (clone 124, 1:50, Dako catalogue number M0887), rabbit–anti-CD103 (clone EPR4166(2), 1:100, Abcam catalogue number ab129202) and rabbit anti-C3 (clone EPR19394, Abcam catalogue number ab200999).

Secondary antibodies: anti-mouse IgG2b AlexaFluor 488 (Invitrogen, A21242), anti-goat Cy2 (Jackson ImmunoResearch Laboratories, catalogue number 705-225-147), anti-rabbit AlexaFluor 488 (Jackson ImmunoResearch Laboratories, catalogue number 111-545-152), anti-mouse IgG1 AlexaFluor 555 (Invitrogen, catalogue number A21127), anti-rat Cy3 (Jackson ImmunoResearch Laboratories, 712-165-153), anti-mouse IgG3 Atto 647 (LifeSpan Biosciences, LS-C209483), anti-chicken AlexaFluor 647 (Invitrogen, catalogue number A21449), AlexaFluor 647 Antibody Labelling Kit (Thermofisher, catalogue number A20186), HRP-labeled goat-anti-rabbit secondary antibody (Dako, K4003), Opal570 Reagent (Akoya, catalogue number OP-001003, used to amplify the signals of anti-pSTAT1). Nuclei were stained with DAPI (Invitrogen, D1306). Secondary antibodies were used at 1:200 dilution.

Of note, neurons were identified using either anti-NeuN or anti-HuC/D antibodies (as specified above) depending on tissue preservation and antigen detectability. HuC/D was applied in cases where NeuN immunoreactivity was insufficient or inconsistent, particularly in tissues with reduced antigenicity due to postmortem delay or fixation variability. Both markers reliably labeled neuronal somata, and selection was made to ensure optimal signal quality for accurate quantification.

### RNAscope in situ hybridization

Fluorescent in situ hybridization (FISH) was done using the RNAscope Fluorescent Multiplex Kit V2 (Cat No.323100). In situ hybridization protocol was performed following recommended specifications for human FFPE brain tissue. Probes against Hs- *ISG15* (Cat No. 467741), Hs-*SNAP25* (Cat No. 518851-C3) and Hs-*B2M* (Cat No. 310161-C2) were commercially available from the supplier.

### High-plex immunofluorescence staining for immune cells infiltrates, neurotoxic astrocytes and oligodendrocytes

Human CNS tissue was fixed in 4% formalin, paraffin-embedded, and sectioned at 2–5 μm thickness onto glass slides. Sections were deparaffinized and subjected to antigen retrieval using a Marmite Pascal system with citrate buffer (pH 6.0). To reduce tissue autofluorescence, slides were incubated in a solution of 4.5% H₂O₂ and 20 mM NaOH in 1 × PBS under LED illumination for two 30/45-min cycles at room temperature (RT).

For the detection of immune cell infiltrates, following a brief incubation in PBS containing 2.5% normal goat serum, sections were incubated for 1 h at RT with a mouse anti-human CD138 antibody (Dako, ref. M7228). Signal detection was performed using an anti-mouse HRP secondary antibody (Dako, ref. K4001) and tyramide signal amplification (TSA Vivid 520, Tocris, ref. 7534). Subsequently, after heat-mediated denaturation (Discovery Roche, CC2), endogenous peroxidase activity was blocked using Dako REAL peroxidase-blocking solution (Dako, ref. K0672), and non-specific binding was prevented with PBS/2.5% goat serum. Sections were then incubated for 1 h at RT with a rabbit anti-human CD4 antibody (Cell Marque, ref. 104R-15), followed by detection using anti-rabbit HRP (Dako, ref. K4003) and TSA Vivid 570 (Tocris, ref. 7535).After a second denaturation step and blocking as described above, sections were incubated overnight at 4 °C with a mouse anti-human CD20 antibody (Leica, ref. NCL-CD20-L26). Signal was visualized using anti-mouse HRP (Dako, ref. K4001) and TSA Opal 780 (Akoya, ref. FP1501001KT). Finally, following another brief blocking step (PBS/2.5% goat serum), sections were incubated for 1 h at RT with a rabbit anti-human CD3 antibody (Dako, ref. A0452), and detection was performed using a donkey anti-rabbit Alexa Fluor 647 secondary antibody (Jackson ImmunoResearch, ref. 712-605-153). Nuclei were counterstained with DAPI (Invitrogen, ref. D1306), and slides were mounted using Fluoromount aqueous mounting medium (Sigma-Aldrich, ref. F4680).

For detection of oligodendrocytes and neurotoxic astrocytes, tissue sections were first incubated briefly in PBS containing 2.5% normal goat serum. Sections were then incubated for 1 h at room temperature (RT) with a rabbit anti-human Olig2 antibody (Roche, ref. 760-5050). Signal was visualized using an anti-rabbit HRP secondary antibody (Dako, ref. K4003) followed by tyramide signal amplification (TSA Vivid 650, Tocris, ref. 7536).Following heat-induced denaturation (Discovery Roche, CC2) and a second blocking step with PBS/2.5% goat serum, sections were incubated for 1 h at RT with a rabbit anti-human C3 antibody (Abcam, ref. ab200999). Detection was achieved using a donkey anti-rabbit Alexa Fluor 488 secondary antibody (Jackson ImmunoResearch, ref. 111-545-152). Subsequently, endogenous peroxidase activity was quenched with Dako REAL peroxidase-blocking solution (Dako, ref. K0672), followed by a third blocking step with PBS/2.5% goat serum. Sections were incubated with an anti-mouse HRP secondary antibody (Dako, ref. K4001), and the signal was amplified using TSA Opal 780 (Akoya, ref. FP1501001KT).Finally, following a brief incubation with 10% normal rabbit serum in PBS, sections were incubated for 1 h at RT with an Alexa Fluor 555-conjugated rabbit anti-human GFAP antibody (Dako, ref. Z0334, labeled using the Alexa Fluor 555 antibody labeling kit, Thermo Fisher, ref. A20187). Nuclei were counterstained with DAPI (Invitrogen, ref. D1306), and slides were mounted using Fluoromount aqueous mounting medium (Sigma-Aldrich, ref. F4680) and acquired with PhenoImager HT 2.0 (Akoya Biosciences).

### Multiplex immunofluorescence staining for brain tissue resident memory T cells

For staining of human fluorescent microscopy sections, a standardized dye cycling method that allows the staining of all five markers (CD8, CD69, BCL2, CD103, GRANZYME B) on the same tissue section was used as previously described [54]. Coverslipped deparaffinized sections were bleached overnight at 4 °C with neutral-white 3-up light-emitting diodes (LEDSupply) to minimize autofluorescence. Coverslips were removed at 50 °C in PBS. Between staining steps, slides were washed with wash buffer (Dako, S3006). Unspecific binding was blocked (PBS/10% FCS) and sections were incubated overnight at 4 °C with rabbit-anti-CD69, mouse IgG2b-anti-CD8 and mouse IgG1-anti-BCL2. Bound primary antibodies were visualized with Alexa Fluor 488 donkey-anti-rabbit (Jackson ImmunoResearch, 111-545-152), Alexa Fluor 555 goat-anti-msIgG1 (Life Technologies, A21127) and AlexaFluor 647 donkey-anti-msIgG2b (Invitrogen, A21242). Nuclei were stained with DAPI (Invitrogen, D1306) and slides were mounted in Fluoromount aqueous mounting medium (Sigma-Aldrich, F4680) for image acquisition. To denature bound antibodies slides were decoverslipped, incubated for 1 h at 50 °C with ULTRA Cell Conditioning Solution (ULTRA CC2, Ventana Medical System, 950-223) and washed in wash buffer (Dako, S3006). Deactivation was verified under the microscope before slides were restained. For restaining, sections were incubated with Dako REAL peroxidase-blocking solution (Dako, K0672), unspecific binding was blocked (PBS/10% FCS) and sections were incubated overnight at 4 °C with mouse IgG2a-anti-GZMB. After incubation with horseradish peroxidase (HRP)-labeled goat-anti-mouse secondary antibody (Dako, K4001), staining was visualized with Opal520 reagent (Akoya, OP-001001). Slides were then stained with rabbit–anti-CD103 and mouse IgG2b-anti-CD8, followed by Alexa Fluor 555 donkey-anti-rabbit (Invitrogen, A31572) and Alexa Fluor 647 donkey-anti-msIgG2b (Invitrogen, A21242). Nuclei were stained with DAPI (Invitrogen, D1306) and slides were mounted in Fluoromount aqueous mounting medium (Sigma-Aldrich, F4680) for image acquisition. Pannoramic Viewer software (3DHISTECH) was used to export TIFF images which were then aligned in Adobe Photoshop CC.

### Quantification of phagocyte engulfing synaptic terminals

For each sample, single-plane confocal images were acquired using a Zeiss LSM800 microscope at 63 × magnification, capturing a total tissue area of 0.06 mm^2^ per image. Image analysis was performed using Imaris software (Bitplane). A dedicated co-localization channel was generated to identify punctate structures positive for both synaptophysin (SYP), a presynaptic marker, and CD68, a lysosomal marker enriched in activated phagocytes. A three-dimensional reconstruction of each IBA1⁺ cell was created to visualize and quantify the presence of internalized synaptic material. Only those phagocytes containing at least one co-localized SYP⁺CD68⁺ puncta fully enclosed within the CD68⁺ cytoplasmic volume were classified as phagocytes actively engulfing synaptic terminals. This conservative criterion ensured internalization rather than surface contact. Quantification was expressed as the number of such SYP⁺CD68⁺ phagocytes per mm^2^ of imaged tissue.

### Quantification of complement C3 deposition at synaptic terminals and phagocyte-mediated engulfment of C3-tagged synapses

For each sample, z-stack of single-plane confocal images were acquired (Zeiss LSM800), sampling 0.06 mm^2^ of tissue at 63 × magnification. Imaris software (Bitplane) was used to build a colocalization channel of synaptophysin (SYP) and C3 positive punctae and to perform a 3D reconstruction of the SYP positive punctae and C3-tagged punctae, therefore calculating a ratio of their volumes to indicate the rate of complement C3 deposition at synapses. IBA1^+^ phagocytes showing colocalized SYP^+^ C3^+^ punctae were counted as phagocytes engulfing C3-tagged synaptic terminals, expressed as number of C3-tagged synaptic terminals /mm^3^ of phagocyte volume.

### Spatial visualization of regional marker density

To visualize the regional density of CD8⁺ T cells, neuronal pSTAT1 expression, and phagocytes engulfing synapses, custom plots were generated using the ggseg package in R (v4.3.1). Marker-specific quantitative data were compiled for selected brain regions and stratified by AE subtype (neuronal surface and intracellular). Data were mapped to anatomical regions of the aseg brain atlas using the ggseg framework. A viridis-based color scale was applied to represent marker intensity with non-evaluated regions indicated in light gray. All data preprocessing and visualization were performed using dplyr, ggplot2, and ggseg.

### Statistical analysis

Data are shown as individual values. Horizontal lines represent median as reported in the figure legends. Normal distribution was confirmed using the D’Agostino–Pearson omnibus normality test where appropriate. To compare two groups, paired non-parametric Wilcoxon test (two-tailed) or Mann–Whitney U non-parametric test was used as indicate in figure legends. Variance between samples was tested using the Brown–Forsythe test. To compare multiple groups with equal variance, one-way ANOVA test was used while Kruskal–Wallis test was applied for groups with unequal variance. Post hoc tests for multiple comparisons are indicated in the figure legends.

In figures, asterisks denote statistical significance as *p < 0.05; **p < 0.01; ***p < 0.001. Statistical analysis was performed in GraphPad Prism9.

### Voxel-based characterization of brain MRI

For a descriptive voxel-based longitudinal analysis of the post-infectious autoimmune encephalitis case, we used the freely available Statistical Parametric Mapping SPM12 package (www.fil.ion.ucl.ac.uk/spm, Wellcome Centre for Human Neuroimaging, UCL London, UK). The MRI data at hand included images at baseline (-11 years before HSV1 encephalitis), at the time of HSV1 encephalitis diagnosis, a follow-up (+ 2 months), at the time of the subsequent post-infectious (PI) NMDAR-antibody AE diagnosis (+ 7 months), and a long-term follow-up (+ 19 and + 32 months).

Based on the fluid-attenuated inversion-recovery FLAIR images at the time of HSV1 encephalitis diagnosis, using histogram-based thresholding, we delineated the lesion volume. This was subsequently used in SPM12s unified segmentation framework[4] as additional subject-specific tissue prior for automated tissue classification of the T1-weighted images in seven tissue classes including grey matter, white matter, cerebrospinal fluid and brain lesion. As additional step, we created two symmetric diffeomorphic registration models [5] to estimate the rate of volume change before and after post-infectious NMDAR-antibody encephalitis including explicitly the number of years between each of the MRI data sets. The resulting Jacobian determinants were multiplied by the volume maps of the mid-point average images to represent the rate of volume change across the two time periods. The grey matter volume changes in brain regions affected and unaffected by FLAIR hyperintensities during HSV1 encephalitis were logarithmically curve-fitted to predict their evolution over time.

### Data resources

The data that support the findings of this study are available from the corresponding authors upon request. All software is freely or commercially available.

## Results

### Neuronal pSTAT1 signatures mark autoimmune encephalitis with antibodies against intracellular antigens

**Fig. 1:**
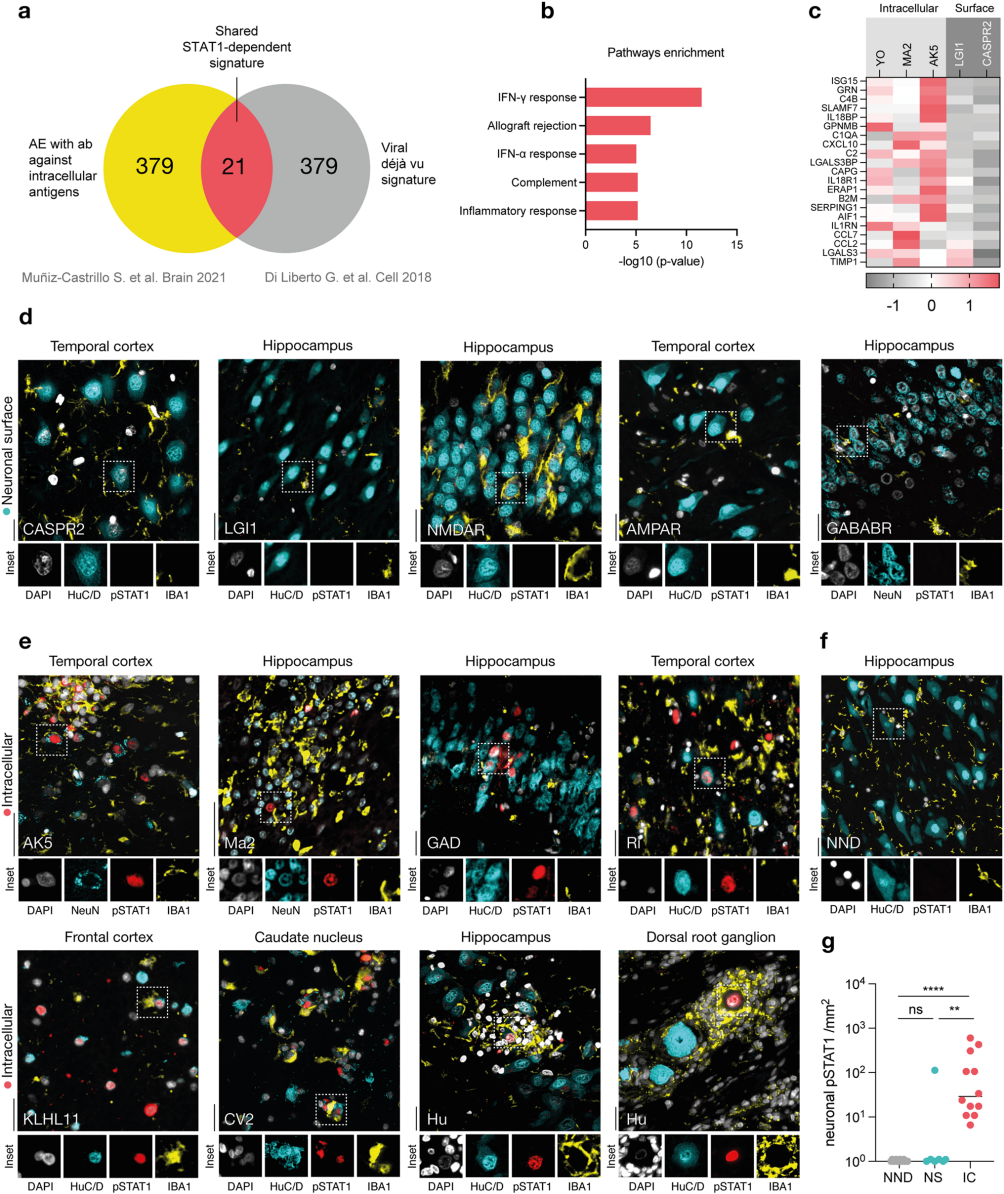
Neuronal pSTAT1 signature in autoimmune encephalitis. **a** Venn diagram showing the overlap between top 400 upregulated transcripts from the neuronal translatome data of the viral déjà vu murine model of T cell-mediated viral encephalitis [14] and the top 400 upregulated cerebrospinal fluid (CSF) proteins from the comparison of autoimmune encephalitis with antibodies against intracellular antigens (IC-AE) and autoimmune encephalitis with antibodies against neuronal surface antigens (NS-AE) [35]. **b** The top five pathways enriched by the shared signature, as identified from the Human Molecular Signatures Database (MSigDB), are presented in descending order based on the −log10 transformation of their p-values. **c** Heatmap displaying the z-score transformed expression levels of proteins from the shared signature in CSF for IC-AE (Yo, Ma2, and AK5) and NS-AE (LGI1 and CASPR2)[35]. **d**–**f** Representative immunostainings for pSTAT1(red), neurons (NeuN or HuC/D, cyan), phagocytes (IBA1, yellow) and nuclei (DAPI, white) in tissue sections of NS-AE (**d**), IC-AE (**e**) and non-neurological disease (NND) controls (**f**). Scale bar = 25 µm. **g** Quantification of neuronal pSTAT1 expression. Data are shown on a base 10 logarithmic scale. Lines indicate the median. Symbols represent individual samples. ****P < 0.0001, *ns* not significant by Kruskal–Wallis test with Dunn’s correction for multiple comparisons

In order to identify common denominators of T cell-mediated encephalitis, we overlapped the top 400 upregulated transcripts from the neuronal translatome data of a murine model of T cell-mediated viral encephalitis [14] with the top 400 upregulated proteins from the CSF of patients suffering from IC-AE [35] (Fig. 1a). This revealed shared predominant IFN-γ-related pathway activation (Fig. 1b) and a distinctive pattern of STAT1-related proteins in the cerebrospinal fluid (CSF) of patients with IC-AE. This distinguished them from those with NS-AE (Fig. 1c), suggesting STAT1 signalling may be pivotal in IC-AE.

To further corroborate this, we performed immunostainings against the activated and phosphorylated form of STAT1 (pSTAT1) in a cohort of brain sections of patients with NS-AE (Fig. 1d, n = 7) and IC-AE (Fig. 1e , n = 12) and NND (Fig. 1f, n = 14) alongside neuronal markers (NeuN or HuC/D) and a phagocyte marker (IBA1) to depict the neuronal apposition of these cells. Neuronal pSTAT1 was primarily detected in IC-AE and was largely absent in NS-AE, except for one case of fulminant encephalitis associated with AMPAR-antibodies (Fig. S1e), thus suggesting that this pathway is rarely activated in NS-AE or NND. Additionally, Hu sensory neuronopathy was associated with increased levels of neuronal pSTAT1 and a high number of phagocytes clustered around pSTAT1-positive neurons in the dorsal root ganglion, showing evidence for this mechanism in both CNS and peripheral nervous system (PNS) neurons. To further investigate downstream targets of STAT1 signaling, we performed RNAscope on tissue sections from NS-AE (n = 5) and IC-AE (n = 11) using probes for *ISG15* and *B2M* (β2-microglobulin) (Fig. S1b), two candidate proteins identified in CSF proteomics (Fig. 1c). Both ISG15 and B2M were more highly expressed in neurons of IC-AE compared to NND (n = 10) (Fig. S1c, d), supporting a neuron-intrinsic interferon response in this AE subtype.

### CD8 + T cells exhibit neuronal apposition and predominantly show a tissue-resident memory phenotype in autoimmune encephalitis

**Fig. 2:**
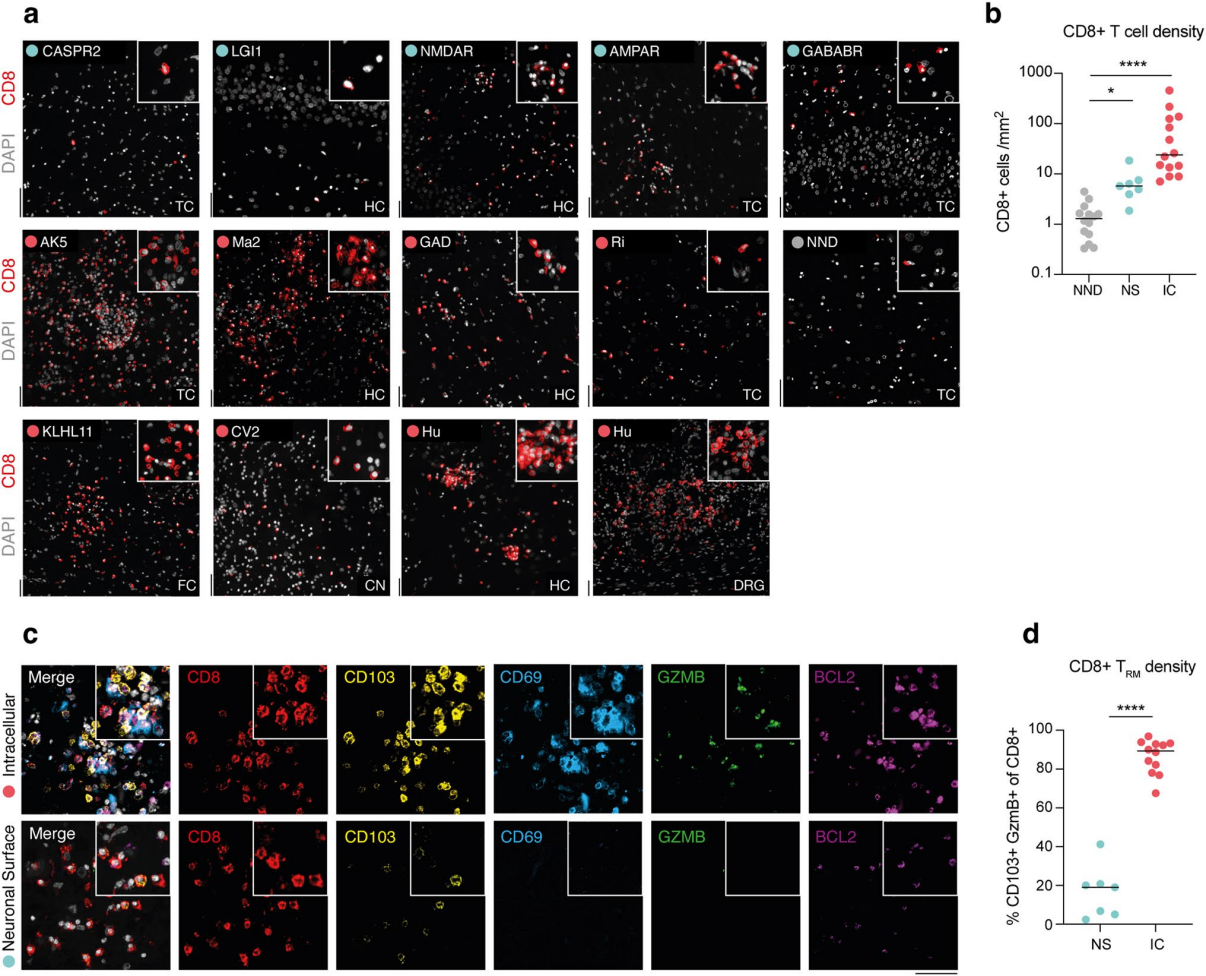
CD8 + T cell infiltration and tissue resident memory signature in autoimmune encephalitis. **a**, **b** Representative immunostainings and quantification of CD8 + T cells (red) in tissue sections with DAPI (white) nuclear counterstaining of NS-AE, IC-AE and NND controls. Regions: temporal cortex (TC), hippocampus (HC), frontal cortex (FC), caudate nucleus (CN), dorsal root ganglion (DRG). **c**, **d** Representative immunostainings and quantification of tissue resident memory (T_RM_) cells co-expressing CD8 (red), CD103 (yellow), CD69 (light blue), GZMB (green) and BCL2 (purple) with DAPI (white) nuclear counterstaining. The proportion of CD103 + GZMB + of CD8 + T cells is shown for NS-AE and IC-AE. Data are shown on a base 10 logarithmic scale (**b**) or standard scale (**d**). Lines indicate the median. Symbols represent individual samples. ****P < 0.0001, *ns* not significant by Kruskal–Wallis test (B) with Dunn’s correction for multiple comparisons and unpaired Student’s *t*-test (D). Scale bar = 50 µm

We hypothesized that neuronal pSTAT1 pathway was induced by IFN-γ from CD8 + T cells [26]. From n = 19 AE cases, we found more CD8 + T cells in the brain parenchyma in IC-AE (84.4 ± 33.2 cells/ mm^2^) compared with infiltrates in either NND (1.5 ± 0.3 cells/ mm^2^) or NS-AE (6.9 ± 2.4 cells/ mm^2^) (Fig. 2a, b).

Multiplex immunofluorescence better characterized the brain infiltrating CD8 + T cells, identifying T_RM_ phenotype in most IC-AE CD8 + T cells (86.4 ± 2.5%) in contrast to few NS-AE (15.7 ± 5.9%) (Fig. 2c, d). These finding suggested a possible role of tissue-resident memory T cells in the pathogenesis of IC-AE.

High-plex immunofluorescence stainings further characterized the immune cell infiltrates (Fig. S2a), revealing increased infiltration of CD3⁺ T cells (Fig. S2b), CD4⁺ T cells (Fig. S2c), and CD20⁺ B cells (Fig. S2d), but not CD138⁺ plasma cells (Fig. S2e), in both IC-AE and NS-AE. Additionally, we observed a higher density of neurons in direct apposition with CD8⁺ T cells in IC-AE compared to NS-AE (Fig. S2f, g).

### Autoimmune encephalitis features neurodegenerative phagocytes engaged in synaptic engulfment and neurotoxic astrocytes

**Fig. 3:**
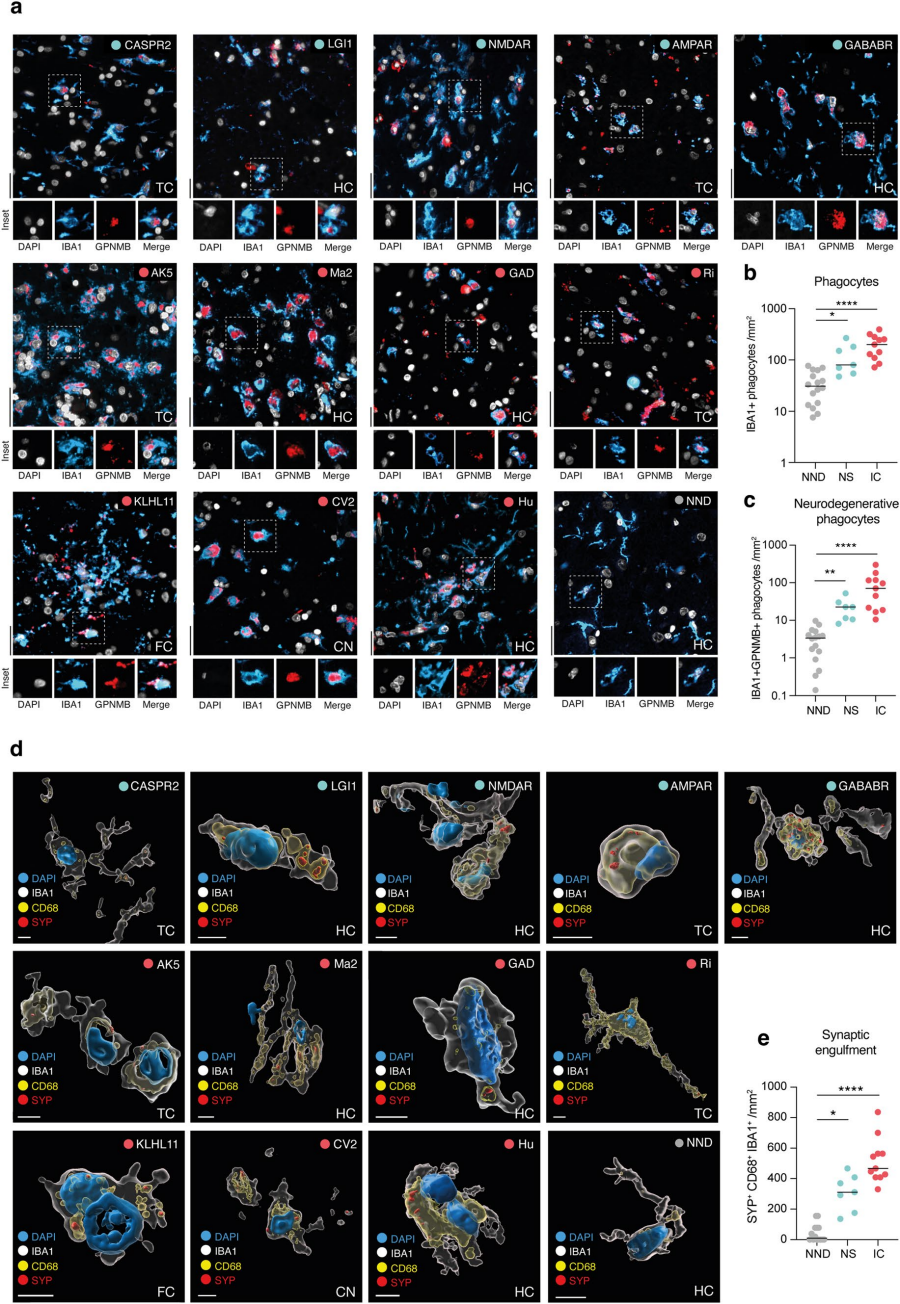
Neurodegenerative phagocytes engulf synapses in autoimmune encephalitis. **a**–**c** Representative immunostainings and quantification of IBA1 + GPNMB + phagocytes in tissue sections with DAPI (white) nuclear counterstaining of NS-AE, IC-AE and NND controls. IBA1 (light blue), GPNMB (red). Data are shown on a base 10 logarithmic scale for tissue densities of IBA1 + phagocytes (**b**) and IBA1 + GPNMB + phagocytes (**c**). Lines indicate the median. Symbols represent individual samples. Regions: temporal cortex (TC), hippocampus (HC), frontal cortex (FC), caudate nucleus (CN), dorsal root ganglion (DRG). **d** 3D cell reconstruction of confocal immunostainings of IBA1 + phagocytes (white) exhibiting synptophysin positive (SYP, red) inclusion overlapping with CD68 + phagosomes (yellow) in NS-AE, IC-AE and NND controls. Nuclei are indicated with DAPI (blue). **e** Quantification of engulfed synaptic terminals (SYP) localized in the phagosomal compartment (CD68) of CNS phagocytes (IBA1) in brain sections of NS-AE (n = 7) and IC-AE patients (n = 11) and NDD (n = 14). Number of SYP + CD68 + phagocytes per mm^2^ are shown. Lines indicate the median. Symbols represent individual samples. ****P < 0.0001, *P < 0.05; ns = not significant by Kruskal–Wallis test with Dunn’s correction for multiple comparisons. Scale bars are 50 µm (A) and 5 µm (D). Regions: temporal cortex (TC), hippocampus (HC), frontal cortex (FC), caudate nucleus (CN)

Reactive phagocytes have been described in AE [6], however their signature markers and role in synaptic engulfment are not known [14]. Here we investigated and compared the phenotypic changes and synaptic loss in NS-AE and IC-AE, finding significantly higher numbers of CNS phagocytes in IC-AE and NS-AE as compared with non-neurological disease controls (NND) (Fig. 3a, b). Furthermore, we stained for GPNMB, a cellular marker that is part of a transcriptional signature in phagocytes associated with neurodegeneration and synaptic engulfment [13], and observed significantly higher numbers (Fig. 3a and c) and proportion (Fig. S3e) of GPNMB-expressing phagocytes in both IC-AE and NS-AE as compared to NND, consistent with more synaptic engulfment in both disease processes (Fig. 3d, e). Astroglial reactivity was assessed by immunostaining for GFAP and complement component C3 (Fig. S3a), a marker associated with neurotoxic astrogliosis (GFAP⁺C3⁺ [29]). In addition, Olig2 + oligodendrocyte density was quantified to evaluate potential alterations in myelinating glial populations in the context of neuroinflammation (Fig. S3a). Both NS-AE and IC-AE showed significantly increased densities of GFAP⁺ astrocytes, neurotoxic astrocytes, and oligodendrocytes compared with NND controls (Fig. S3b–d), indicating a common glial response across autoimmune encephalitis subtypes.

### Regional enrichment of CD8 + T cells aligns with neuronal pSTAT1 expression and synaptic engulfment

Although tissue availability varied across brain regions, analysis of the available samples indicated that CD8 + T cell infiltration was not uniformly distributed across brain areas within either subtype. Higher densities were consistently found in the frontal cortex and hippocampus compared to other regions in IC-AE (Fig. S4a). Notably, brain regions with higher CD8 + T cell infiltration also exhibited increased neuronal pSTAT1 expression (Fig. S4b) and enhanced synaptic engulfment (Fig. S4c). Furthermore, the topographic distribution of CD8 + T cell clusters closely mirrored that of neuronal pSTAT1 in highly infiltrated regions (Fig. S4d–f), indicating a spatial coupling between CD8 + T cell infiltration and activation of the neuronal pSTAT1 pathway.

### Complement C3 deposition at synapses and in engulfed phagocytes prevails in autoimmune encephalitis with antibodies against neuronal surface antigens

Previous experimental studies indicated that the classical complement cascade can be activated at the synapses, resulting in the deposition of C1q and downstream C3 promoting synaptic engulfment via CNS phagocytes expressing the receptor for C3 (CR3) [53, 57]. Yet, synaptic engulfment can also occur independently of C3 during neuroinflammation [14].

**Fig. 4:**
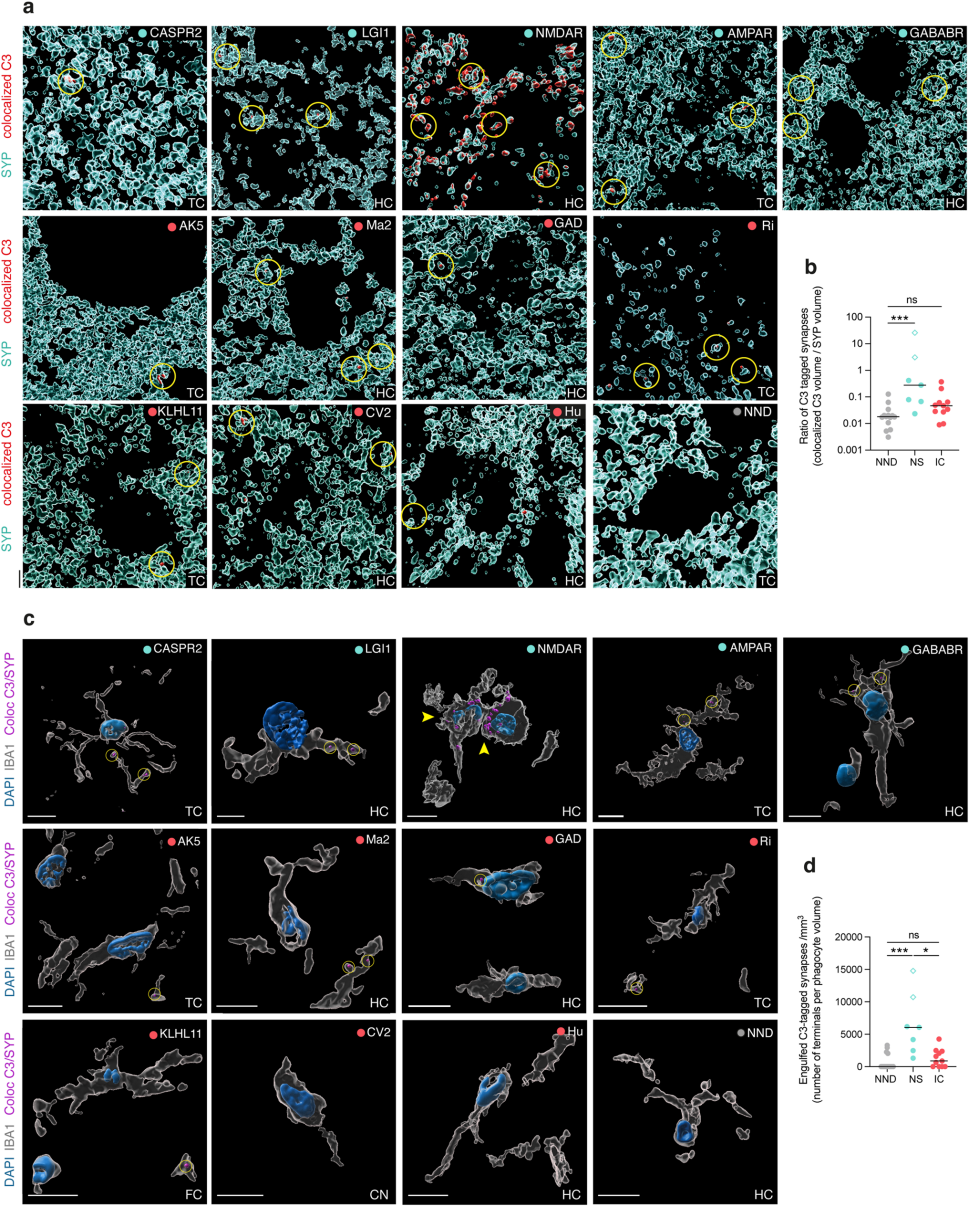
Complement C3 deposition at synaptic terminals and engulfment of C3-tagged synapses in autoimmune encephalitis. **a** 3D reconstruction of confocal immunostainings of synaptophysin (SYP, cyan) and complement C3 colocalization with synaptic terminals (C3, red) in brain sections of NS-AE, IC-AE and NND controls. Yellow circles highlight C3 deposition at synapses. **b** Ratio of C3-tagged synapses indicated as the proportion of the colocalized C3 volume over the total SYP volume for NS-AE (n = 7), IC-AE (n = 11) and NND controls (n = 14). Data are shown on a base 10 logarithmic scale, symbols represent individual samples. NMDAR-antibody AE is indicated by a diamond shape. **c** 3D cell reconstruction of confocal immunostainings of IBA1 + phagocytes (white) exhibiting C3-tagged synapses (C3-SYP, purple) inclusion in NS-AE, IC-AE and NND controls. Nuclei are indicated with DAPI (blue). **d** Quantification of engulfed C3-tagged synaptic terminals (SYP) of CNS phagocytes (IBA1) in brain sections of NS-AE (n = 7) and IC-AE patients (n = 11) and NDD (n = 14). Number of engulfed C3-SYP + terminals per phagocytes (mm^3^) are shown. NMDAR-antibody AE is indicated by a diamond shape. ***P < 0.001, **P < 0.01, *P < 0.05; ns = not significant by Kruskal–Wallis test with Dunn’s correction for multiple comparisons. Scale bar = 5 µm. Regions: temporal cortex (TC), hippocampus (HC), frontal cortex (FC), caudate nucleus (CN)

To evaluate the spatial association of complement C3 deposition on synapses in human AE, we immunostained brain sections from patients with NS-AE, IC-AE, and NND, colocalising synaptophysin (SYP) with C3, to quantify the extent of C3-bound synapses. In NS-AE, we found significantly more C3 deposition at synapses, especially in NMDAR encephalitis, than in NND, which was distinct from IC-AE (Fig. 4a, b). This increased C3 deposition correlated with significantly more engulfed C3-tagged synapses by CNS phagocytes in NS-AE as compared to NND and IC-AE (Fig. 4c, d), suggesting that synapses tagged with C3 represent a distinct pathophysiological feature in NS-AE.

### Atrophic changes and neuronal pSTAT1 expression in post-infectious autoimmune encephalitis

Given that patients with herpes simplex virus (HSV) encephalitis can develop post-infectious autoimmune encephalitis [3], usually with a poor outcome, we asked whether the histopathological hallmarks aligned more with NS-AE or IC-AE. We examined a case of a patient who developed NMDAR encephalitis 7 months after the initial HSV1 infection. The patient received immunotherapeutic interventions, including intravenous immunoglobulins (IVIg) at diagnosis and subsequent biannual administrations of rituximab (RTX). Despite these measures, the MRI follow-up indicated extensive brain atrophy accompanied by important neuropsychological sequelae observed on the clinical ground (Fig. S5a).

To quantify the extent of this atrophy, we employed a voxel-based longitudinal analysis of MRI images to delineate the brain regions affected by HSV1-associated FLAIR hyperintensities (Fig. S5bB) and to track the progression of atrophy before (Fig. S2c) and after the diagnosis of post-infectious AE (Fig. S5d). Remarkably, over a period of 32 months following HSV1 encephalitis, we observed progressive atrophic changes (Fig. S5e) in both the CNS regions that were and were not affected by FLAIR hyperintensities at the time of the HSV1 encephalitis diagnosis.

**Fig. 5:**
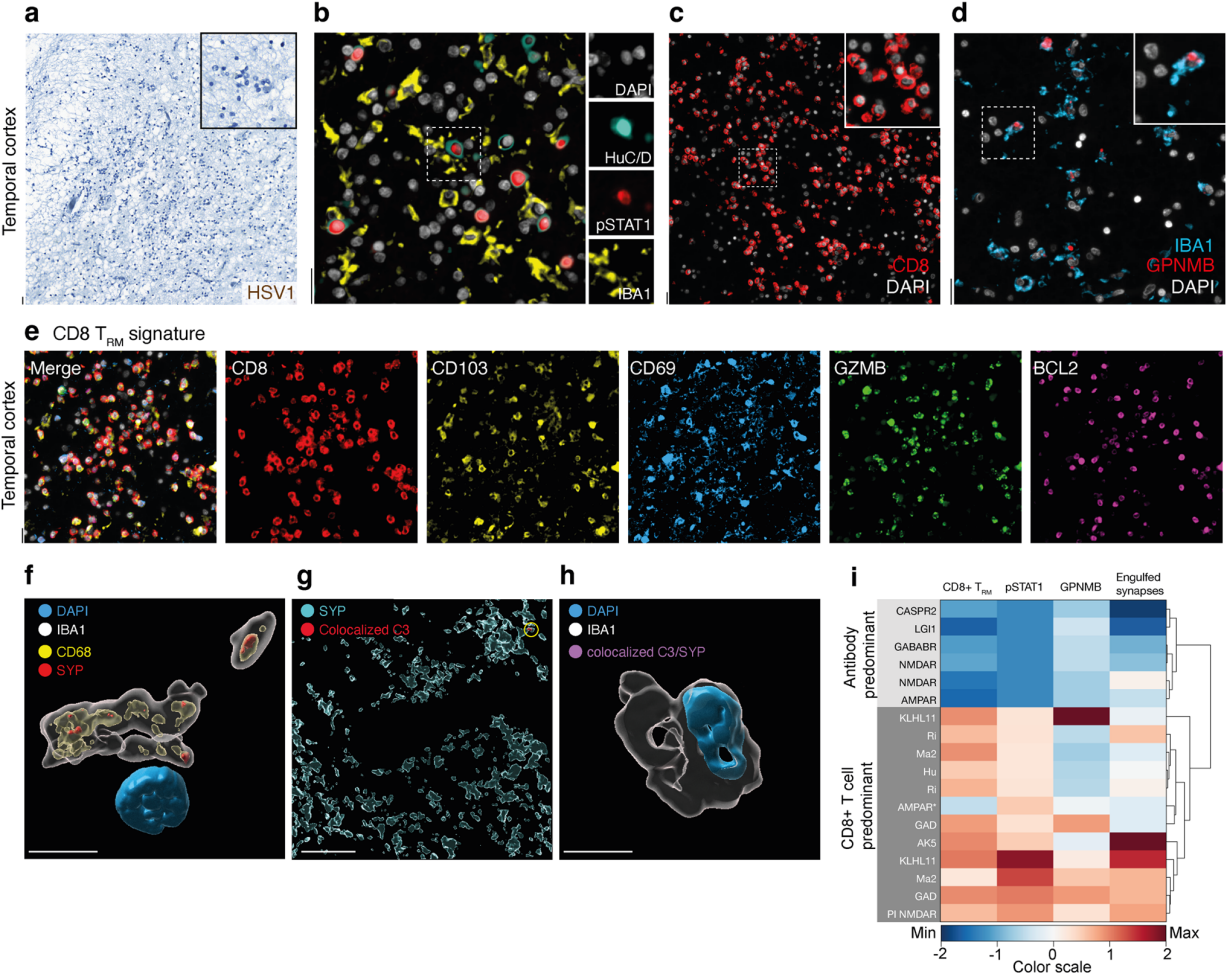
Pathological insights in post-infectious autoimmune Encephalitis. **a** Representative immunohistochemistry for HSV1 (DAB, brown) in PI NMDAR-antibody encephalitis indicating the absence of infection. **b** Representative immunostaining for pSTAT1(red), neurons (HuC/D, cyan), phagocytes (IBA1, yellow) and nuclei (DAPI, white) in tissue sections of PI NMDAR-antibody encephalitis. **c** Representative immunostaining of CD8 + T cells (red) with DAPI (white) nuclear counterstaining in tissue sections of PI NMDAR-antibody encephalitis. **d** Representative immunostainings of IBA1 + GPNMB + phagocytes with DAPI nuclear (white) counterstaining of post-infectious NMDAR-antibody encephalitis. IBA1 (light blue), GPNMB (red). **e** Representative immunostainings of tissue resident memory (T_RM_) cells co-expressing CD8 (red), CD103 (yellow), CD69 (light blue), GZMB (green) and BCL2 (purple) with DAPI (white) nuclear counterstaining in PI NMDAR-encephalitis. **f** 3D cell reconstruction of confocal immunostainings of IBA1 + phagocytes (white) exhibiting synaptophysin positive (SYP, red) inclusion overlapping with CD68 + phagosomes (yellow) in PI anti-NMDAR encephalitis. **g** 3D reconstruction of confocal immunostainings of synaptophysin (SYP, cyan) and complement C3 colocalization with synaptic terminals (C3, red) in brain sections of PI NMDAR-antibody encephalitis. **h** 3D cell reconstruction of confocal immunostainings of IBA1 + phagocytes (white) showing the absence of inclusions of C3-tagged synapses (C3-SYP, purple) in PI NMDAR-antibody encephalitis. **i** Heatmap and dendrogram analysis of CD8 + T_RM_ cells, neuronal pSTAT1 levels, GPNMB + phagocyte densities, and synaptic engulfment different autoimmune encephalitis samples. PI NMDAR and fulminant AMPAR-antibody AE (AMPAR*) cluster with IC-AE. The color scale represents minimum and maximum values, which are determined after scaling each variable by columns. For pSTAT1, a signal absence is assigned a value of −2. Positive values range from 0.01 to 2 after scaling. Scale bars: 20 µm (**a**–**e**, **g**) and 5 µm (**f**, **h**)

Although, immunohistochemistry confirmed the absence of HSV1 in the brain tissue upon autopsy (36 months later than HSV1 encephalitis) (Fig. 5f), we observed a robust neuroinflammatory response characterized by a prominent neuronal expression of pSTAT1 (Fig. 5a), infiltrates of CD8 + T cells and accumulation of GPNMB + phagocytes (Fig. 5b–d).

Co-immunostaining for CD8, CD103, CD69, BCL2 and GZMB showed that infiltrating CD8 + T cells expressed markers compatible with T_RM_ signature (Figs. 5e), like those seen in other forms of T cell-mediated encephalitis (Fig. 2c, d). This finding is crucial as it suggests the persistence of CD8 + T_RM_ within the brain, potentially contributing to sustained immune activation and tissue damage following HSV1 infection.

Additionally, we found phagocytes engulfing synapses without complement component C3 tagging (Fig. 5f–h). These neuropathological features together underscore a predominant T cell mediated pathogenesis in post-infectious autoimmune encephalitis. This was supported by the clustering of these features (Fig. 5i) with IC-AE and other severe forms of encephalitis, such as fulminant AMPAR-antibody encephalitis (AMPAR*), which also showed high levels of neuronal pSTAT1 expression (Fig. S1e), thus suggesting a shared pathway involving CD8 + T_RM_ cells in the pathogenesis of these debilitating neurological conditions.

### Immunotherapy-associated modulation of immune and glial markers in AE

Although the small sample size following stratification by treatment and AE subtype precluded rigorous statistical comparisons, exploratory examination of Z-score-normalized heatmaps indicated potential treatment-associated modulation of histopathological markers in both NS-AE and IC-AE (Fig. S5f–g). Overall, despite immunotherapy, IC-AE cases continued to exhibit greater immune cell infiltration, pSTAT1 activation, phagocyte accumulation, and synaptic loss compared to NS-AE. However, treatment with corticosteroids or rituximab—administered alone or in combination—was associated with lower levels of T and B cell infiltration, GPNMB⁺ phagocytes, and GFAP⁺C3⁺ neurotoxic astrocytes in IC-AE (Fig. S5f). In contrast, T_RM_ cell levels appeared largely unaffected by any of the treatment modalities evaluated. Notably, neuronal pSTAT1 expression and GPNMB⁺ cell density appeared reduced in cyclophosphamide-treated cases.

To explore the potential impact of treatment intensity, we also assessed histopathological profiles in relation to the number of immunotherapy lines (Fig. S5g). A higher number of treatment lines (≥ 3) was associated with reduced expression of immune and neuroinflammatory markers in both NS-AE and IC-AE. In IC-AE, this trend was most apparent for pSTAT1 expression, which was lower in more extensively treated cases. Conversely, in NS-AE, minimal or no treatment (0–1 lines) correlated with elevated C3-tagging. These findings suggest that intensified immunosuppressive therapy might mitigate certain neuroinflammatory features, especially in IC-AE, but also highlight that current therapeutic strategies may not sufficiently reduce T_RM_ cell accumulation in the CNS.

## Discussion

Understanding the mechanisms underlying immune-mediated neuronal damage to neurons in AE is paramount for diagnostic biomarker development and guides clinicians toward more targeted therapy management of affected patients [15]. It furthermore sheds light on the functional basis behind the enduring cognitive deficits and epilepsy seen in a subgroup of affected individuals [23, 34].

In our previous study, we have shown that neuronal signaling through STAT1 is implicated in phagocyte-mediated synaptic elimination following CD8 + T cell attacks in the murine model of viral encephalitis [14], thus suggesting a pivotal role in orchestrating immune-mediated neurodegeneration in human neuroinflammatory disorders.

In this largest histopathological cohort of AE cases to date, the current study presents evidence of neuronal pSTAT1 as a promising biomarker to distinguish IC-AE and NS-AE, particularly in cases where the disease-driving antigens remain unidentified. Given that a substantial portion of AE patients do not exhibit detectable autoantibodies [12, 28], the detection of STAT1-related molecules in cerebrospinal fluid (CSF), such as ISG15, B2M or IL18BP [35], may offer valuable insights into distinguishing prevailing immunopathogenic mechanisms, independently of identifying anti-neural antibodies. Additionally, the identification of pSTAT1 as a critical player in the pathogenesis of AE may guide the development of targeted therapies aimed at modulating the pSTAT1 pathway, potentially leading to improved outcomes for IC-AE patients [9].

In line with previous findings [6], the infiltration of CD8 + T cells into the brain parenchyma is a notable feature of IC-AE, setting it apart from NS-AE. Even more intriguing is the qualitative difference observed in these infiltrates, with a larger proportion of CD8 + T cells in IC-AE acquiring a tissue-resident memory phenotype (T_RM_). This observation aligns with current concepts suggesting that a compartmentalized immune response involving long-lived T_RM_ cells may be responsible for perpetuating tissue damage, as previously shown in paraneoplastic encephalitis [18]. One mechanism by which CD8 + T_RM_ cells contribute to damage is through the production of IFN-γ. This cytokine enhances the phagocytic abilities of microglia, which in turn promotes the engulfment of synapses in murine models of neurotropic flavivirus infections [19].

Here, we show that during AE, CNS phagocytes exhibit elevated expression levels of GPNMB, a surface marker previously recognized as a constituent of a microglial signature enriched in neurodegenerative diseases [17, 44]. The elevated numbers of CNS phagocytes, coupled with their increased expression of GPNMB suggest a link between these cells and the pathogenesis of AE.

In a previous study, we have shown that GPNMB + phagocytes are involved in synaptic elimination in Neuro-HIV [14]. Consistent with this notion that synaptic pathology may contribute to the development of cognitive sequelae, we provide here evidence that CNS phagocytes engulf synaptic terminals in AE, a feature that appears to be more pronounced in IC-AE with a usually worse clinical course than NS-AE.

Furthermore, our study indicates that the role of complement C3 in synaptic engulfment appears to differ between NS-AE and IC-AE, with a marked presence of complement C3 at synapses in NS-AE, and particularly in NMDAR-antibody encephalitis [50], in which complement-fixing IgG1 antibodies are generally more prevalent compared to those found in LGI1 [48] or CASPR2- antibody AE[52]. Additionally, C3-tagged synapses are more abundantly observed inside the CNS phagocytes of NS-AE, pointing towards a complement-mediated mechanism for synaptic engulfment.

Previous investigations into the role of complement in the pathogenesis of autoimmune encephalitis primarily concentrated on the terminal components of the complement cascades, notably C9neo, which forms a part of the lytic pathway and the membrane attack complex (MAC) [6, 25]. However, these studies could not detect its deposition in brain sections of individuals with NMDAR antibody encephalitis [32, 63]. It’s worth noting that these earlier investigations did not specifically assess the deposition of the initial components of the complement cascade, such as C1q and C3, at the synaptic level. These components are crucial for the complement tagging of synapses and their subsequent engulfment mediated by CNS phagocytes via CR3. In contrast, in IC-AE, C3 deposition is less prominent and thus, complement-independent mechanisms such as local externalization of phosphatidylserine [37] or the activation of JAK2-STAT1 pathway in neurons and elimination of weakened inactive synapses [14, 61] are likely crucial in orchestrating phagocyte-mediated synaptic loss in such cases.

We observed a notable increase in GFAP⁺C3⁺ astrocytes in AE, indicative of a reactive astrocyte phenotype characterized by upregulation of complement component C3. This finding aligns with a previous study demonstrating that neuroinflammatory conditions can induce neurotoxic astrocytes expressing high levels of C3, which may contribute to neuronal damage and synaptic dysfunction [29].

Additionally, our analysis revealed elevated numbers of Olig2⁺ cells in AE. Olig2 is a transcription factor critical for the development and differentiation of oligodendrocyte precursor cells (OPCs). The increased presence of Olig2⁺ cells may reflect a reactive response to oligodendrocyte injury [56], although oligodendrocyte abundance may vary with the anatomical region analyzed. Moreover, our study introduces a novel aspect to the immunopathology of post-infectious autoimmune encephalitis by emphasizing the involvement of CD8 + T cells, a role not previously recognized in this disease context. The initial neuronal damage caused by HSV infection may act as a catalyst for an autoimmune cascade targeting a wide array of neuronal antigens [10] promoting the development and persistence of autoreactive CD8 + T_RM_ cells within the brain. The sustained presence of these T_RM_ cells is of particular concern because they can continue to exert pathogenic effects long after the initial infection has resolved [18]. These cells likely drive ongoing grey matter atrophy via activation of the neuronal STAT1-signaling pathway and could potentially contribute to the chronicity and severity of the disease. This might be peripherally reflected by the presence of a higher interferon signature in the blood of patients with post-HSV AE as compared with patients with HSV-encephalitis who did not develop AE [2]. Of note, several additional mechanisms could contribute to the atrophic changes observed in follow-up MRI scans after the diagnosis of post-infectious autoimmune encephalitis (AE). These include Wallerian degeneration, neuronal damage induced by viral proteins [31] and autoantibody-mediated damage [21].

The classification of autoimmune encephalitis (AE) into categories based on antigen location (surface vs. intracellular) carries profound implications for clinical practice. Nonetheless, autoantibodies are not identified in approximately one-third of AE cases [20], complicating both diagnosis and the selection of an effective immunotherapeutic strategy. Here, we observed abundant CD8 + T_RM_ cell infiltrates and a prominent neuronal pSTAT1 signature in a severe case of AMPAR-antibody encephalitis and post-infectious NMDAR-antibody encephalitis. This finding suggests that, in addition to autoantibodies, T cells targeting alternative antigens may play a role in exacerbating the severity of these disorders. Notably, immunohistochemical analyses of brain tissue, serum, and cerebrospinal fluid from patients with post-HSV AE reveal the development of multiple autoantibodies, though not all antigenic targets have been identified [3]. It is postulated that antigens from neurons damaged by the virus could trigger an autoimmune response [10].

These findings suggest that existing therapeutic strategies primarily focusing on B cells may fall short for such conditions. The expanded understanding of T cell involvement in post-infectious autoimmune encephalitis not only enriches our comprehension of the disease’s pathogenesis but also sets the stage for innovative approaches to its treatment by taking advantage of the neuronal pSTAT1 signature.

While this study provides valuable insights into the immunopathogenesis of AE, it is essential to acknowledge its limitations. In terms of the complement-cascade, our focus was restricted on complement component C3, but it’s possible that other components of the complement system also play significant roles in tagging synapses for phagocyte-mediated engulfment.

Despite immunosuppressive therapy, IC-AE and NS-AE maintained their distinct immunopathological features, with variable modulation across different treatment regimen. While increased immunotherapy exposure appeared to attenuate certain immune and neuroglial markers—particularly in IC-AE—no therapy seems to efficiently diminish the abundance of T_RM_, suggesting potential resistance to current treatment approaches. Importantly, individuals receiving multiple lines of immunotherapy often still exhibited substantial immunopathological burden, especially in IC-AE, likely reflecting more aggressive disease courses.

Furthermore, while our study assembled a considerable number of AE cases, the overall cohort size and clinical heterogeneity among AE subtypes represent still a limitation which warrants further investigations. Notably, a high proportion of NS-AE patients in our series died within a few months of symptom onset. This unusually rapid progression contrasts with the typically more favorable prognosis of NS-AE [51] and may reflect a selection bias inherent to autopsy-based studies, where severe or treatment-refractory cases are overrepresented. These factors underscore the need for even larger, prospective cohorts and mechanistic investigations to validate and expand upon the present findings.

In summary, the current study expands our understanding of the underlying mechanisms of neuronal damage in AE and suggests a histopathological classification of CNS lesions in AE. Patients with immune responses against intracellular antigens feature neuronal pSTAT1 and predominant T_RM_ cells infiltrates, which together with neurodegenerative phagocytes, contribute to synaptic pathology. In contrast, complement deposition at synapses and their engulfment by phagocytes appears to be a more dominant feature of NS-AE. A segregation of AE based on the prevailing disease pathogenesis of CNS lesions may have implications for patient management, where interdisciplinary approach involving neurology, immunology, and neuropathology could enable tailored treatments in the future of individualized patient care.

## Supplementary Information

Below is the link to the electronic supplementary material.

Supplementary file1 (DOCX 31 KB)

Supplementary file2 (DOCX 19 KB)

Supplementary file3 (TIF 10372 KB) Figure S1 – Comparative analysis of brain regions and immune markers in autoimmune encephalitis with multiplex fluorescence in situ hybridization and immunostaining. (a) Age-matched AE cases and non-neurological disease (NND) controls in cortical regions such as temporal cortex / hippocampus (red) and frontal cortex (light blue) and non-cortical regions such as basal ganglia (orange) and brainstem (green). Symbols represent individual samples, squares indicate male sex, circles indicate female sex. (b) Representative RNAscope fluorescence in situ hybridization images from NS-AE, IC-AE, and NND brain sections showing co-detection of ISG15 (magenta), B2M (yellow), and the neuronal marker SNAP25 (cyan), with DAPI counterstain (white). (c-–d) Quantification of ISG15 (c) and B2M (d) positive punctae localized within SNAP25-positive neuronal areas. (e) Multiplex immunostaining for pSTAT1(red), neurons (NeuN, cyan), phagocytes (IBA1, yellow) and nuclei (DAPI, white) in tissue sections of fulminant AMPAR-antibody AE (AMPAR*). ***P < 0.001, **P < 0.01, *P < 0.05; ns = not significant by Kruskal-Wallis test with Dunn’s correction for multiple comparisons. Scale bar = 20 µm. Regions: temporal cortex (TC), hippocampus (HC), frontal cortex (FC), caudate nucleus (CN).

Supplementary file4 (TIF 8755 KB) Figure S2 – Profiling of immune cell infiltrates and contact of CD8+ T cell with neurons in autoimmune encephalitis. (a–d) Representative immunofluorescence images (a) and corresponding quantification (b–d) of brain sections stained for CD3 (white), CD4 (red), CD20 (yellow), CD138 (cyan), and nuclei (DAPI, blue) across the indicated groups. (f–g) Representative images (f) and quantification (g) of sections stained for HuCD (red, neuronal marker), CD8 (yellow), and DAPI (nuclei), highlighting direct contacts between CD8⁺ T cells and neurons. Quantification in (g) shows the density of HuCD⁺ neurons in contact with CD8⁺ T cells. Data are shown on a base 10 logarithmic scale (b-d) or standard scale (g). Lines indicate the median. Symbols represent individual samples. ***P < 0.001, **P < 0.01, *P < 0.05; ns = not significant by Kruskal-Wallis test with Dunn’s correction for multiple comparisons. Scale bar = 50 µm. Regions: temporal cortex (TC), hippocampus (HC), frontal cortex (FC), caudate nucleus (CN), dorsal root ganglion (DRG).

Supplementary file5 (TIF 12383 KB) Figure S3 – Reactive gliosis in autoimmune encephalitis. (a–d) Representative immunofluorescence images (a) and corresponding quantifications (b–e) of brain sections stained for GFAP (cyan), C3 (red), Olig2 (yellow), and nuclei (DAPI, white) across the indicated experimental groups. (c) Neurotoxic astrocytes are identified as GFAP⁺/C3⁺ double-positive cells. (d) Oligodendrocytes are identified by Olig2 positivity. (e) Quantification of brain sections stained for IBA1, GPNMB, and DAPI, highlighting the proportion of phagocytic cells expressing the neurodegenerative marker GPNMB. Data are shown on a base 10 logarithmic scale (b-d) or standard scale (e). Lines indicate the median. Symbols represent individual samples. ***P < 0.001, **P < 0.01, *P < 0.05; ns = not significant by Kruskal-Wallis test with Dunn’s correction for multiple comparisons. Scale bar = 25 µm. Regions: temporal cortex (TC), hippocampus (HC), frontal cortex (FC), caudate nucleus (CN).

Supplementary file6 (TIF 6745 KB) Figure S4 - Regional distribution of CD8⁺ T cell infiltration, neuronal pSTAT1 activation, and synaptic pathology. (a–c) Quantification of CD8⁺ T cell density (a), neuronal pSTAT1⁺ cells (b), and synaptic engulfment by phagocytes (IBA1⁺SYP⁺CD68⁺) (c) in the frontal cortex, hippocampus/temporal cortex, caudate, and brainstem of NS and IC groups. The color scale denotes the severity of histopathological alterations across regions. (d-f) Representative low-magnification immunofluorescence images illustrating the distribution of CD8⁺ T cells and neuronal pSTAT1 expression in brain sections of frontal cortex (d), hippocampus (c) and caudate nucleus (e) stained for CD8 (yellow), pSTAT1 (red), and HuC/D (light blue, neuronal marker). Scale bar = 500 µm. Data are shown on a base 10 logarithmic scale (a-c). Lines indicate the median. Symbols represent individual samples. **P < 0.01, *P < 0.05; comparisons between NS and IC in the hippocampus and temporal cortex were performed using the Mann–Whitney U non-parametric test.

Supplementary file7 (TIF 4996 KB) Figure S5 – Neurodegenerative sequelae in post-infectious autoimmune encephalitis and impact of immunotherapies on histopathological markers. (a) Disease Progression Timeline: MRI scans depicting disease progression in a patient with post-infectious autoimmune encephalitis, including initial baseline (-11 years), HSV1 encephalitis diagnosis, follow-up (+2 months), subsequent post-infectious (PI) NMDAR-antibody encephalitis diagnosis (+7 months), and long-term follow-up (+19 and 32 months) culminating in an autopsy 36 months after the diagnosis of HSV1 encephalitis. (b) 3D volume rendering of FLAIR hyperintensities during HSV1 encephalitis overlayed on the brain template. (c) Atrophic changes during the interval between the diagnosis of HSV1 encephalitis and post-infectious AE are depicted on the brain template. (d) Atrophic changes after the diagnosis of post-infectious AE are depicted on the brain template. (e) Progressive grey matter (GM) volume changes in regions initially affected and non-affected by FLAIR hyperintensities at the time of HSV1 encephalitis diagnosis are logarithmically curve-fitted to predict their evolution over time. (f–g) Heatmaps display the effect of immunotherapy (f) and number of immunotherapy lines (g) on histopathological markers in neuronal surface (NS) and intracellular (IC) autoimmune encephalitis (AE), presented as Z-scores. (f) Impact of specific immunotherapies (administered alone or in combination) on histopathological markers, stratified by NS and IC subtypes. Immunotherapies include intravenous immunoglobulins (IVIg), plasmapheresis (PLEX), corticosteroids, cyclophosphamide, and rituximab. (g) Association between the number of immunotherapy lines (0–1, 2, or ≥3) and histopathological marker expression in NS and IC AE subtypes. Histopathological markers include T and B cell markers (CD4, CD8, CD20, CD138), tissue-resident memory CD8⁺ T cells (TRM), interferon signaling (pSTAT1, ISG15, B2M), markers of neurotoxic astrocytes (GFAP⁺ C3⁺), neurodegenerative phagocytes (GPNMB), and immunopathological interactions (CD8–neuron contact, C3-tagging, engulfed synapses). Color scale represents Z-scores, ranging from low (blue) to high (red), indicating relative expression across treatment groups.
