# Supplementary material for: Neuronal pSTAT1 hallmarks synaptic pathology in autoimmune encephalitis against intracellular antigens
Source: Acta Neuropathol. 2025 Apr 25;149(1):35. doi: 10.1007/s00401-025-02882-7 (PMC12031792; doi:10.1007/s00401-025-02882-7)
Supplement: Supplementary file 1 — Supplementary file1 (DOCX 31 KB) [file 401_2025_2882_MOESM1_ESM.docx]

**Table S1 – Autoimmune encephalitis cases**

**Autoimmune encephalitis against neuronal surface antigens**

| **Case ID** | **Disease** | **Antibody titer (if available)** | **Type of sample** | **Age at biopsy/**  **autopsy** | **Sex** | **CNS region** | **Disease duration** | **Cancer association** | **Clinical data** | **Imaging abnormalities** | **Treatment** | **Cause of death (if autopsy** |
| --- | --- | --- | --- | --- | --- | --- | --- | --- | --- | --- | --- | --- |
| CASPR2_1 | anti-CASPR2 encephalitis | Serum 1:30000; CSF 1:500 | FFPE, autopsy | 77 | M | Temporal and hippocampus | 3 years | No | Seizures, behavioral abnormalities, memory impairment. | Normal MRI | Steroids and PLEX | SUDEP in context of CASPR2 relapse |
| GABABR_1 | anti-GABABR encephalitis | Positive in CSF by IHC on tissue and CBA | FFPE, autopsy | 60 | M | Temporal and hippocampus | 1 month | Yes (SCLC) | Limbic encephalitis: Psychiatric and sleep disturbances, then seizures. | Bilateral amygdalo-hippocampal thickening with pronounced right-side FLAIR T2 hypersignal extending to the insular cortex and parahippocampal gyrus | Steroids,  IVIg | Pneumonia |
| AMPAR_1 | anti-AMPAR encephalitis | Positive in CSF by IHC on tissue and CBA | FFPE, autopsy | 51 | M | Frontal | 1 month | No | Fulminant encephalitis with cerebellar syndrome and right face hypoesthesia, then seizures and  coma. | Multiple flaky T2 hypersignals predominantly in insula and posterior cerebral fossa | Steroids,IVIg and cyclophosphamide | Cardiorespiratory arrest with extensive cortical necrosis due to encephalitis |
| AMPAR_2 | anti-AMPAR encephalitis | Serum 1:1600; CSF 1:32; tissue based assay on prefixed rat brain and in-house CBA | FFPE, autopsy | 69 | F | Temporal / hippocampus | 3.5 months | Yes (lung adenocarcinoma) | Acute global amnesia | Bilateral hippocampal FLAIR/T2 hyperintensities | Intravenous steroids, PLEX, chemotherapy; partial response after PLEX | Tumor progression |
| LGI1_1 | Anti-LGI1 | Positive in serum and CSF | FFPE, autopsy | 80 | M | Temporal / hippocampus | Not available | No | Not available | Not available | Not available | Not available |
| NMDAR_1 | anti-NMDAR encephalitis | Serum 1:1000; CSF 1:100 | FFPE, autopsy | 28 | M | Temporal and hippocampus | 2 months | No | Psychiatric manifestation, cognitive abnormalities, dysautonomia. | Normal MRI | No immunotherapy | Intensive therapy Unit complications |
| NMDAR_2 | anti-NMDAR encephalitis | Positive in serum and CSF | FFPE,  biopsy | 16 | F | Temporal lobe | 3 months | No | Behavioural symptoms, seizures, movement disorder. | Normal MRI | Intravenous steroids | None (biopsy) |
| NMDAR_3 | Post-infectious Anti-NMDAR encephalitis | CSF 1:16; absent in serum | FFPE, autopsy | 50 | F | Temporal | 3 years | No | post-infectious (HSV1) anti-NMDAR encephalitis with new onset of behavioral disturbance (alcohol consumption and hyperphagia), insomnia, and aggravation of cognitive symptoms. | At the time of AE diagnosis: extensive cystic encephalomalacia in the left temporal region. Additionally, notable volume loss in the bilateral temporal areas, left occipital, left fronto-parietal, and right frontal regions. | IVIg, rituximab | Cardiorespiratory arrest |

Abbreviations: cerebrospinal fluid (CSF), intravenous immunoglobulin (IVIg), plasma exchange (PLEX), small cell lung cancer (SCLC)

**Autoimmune encephalitis against intracellular antigens**

| **Case ID** | **Disease** | **Antibody titer (if available)** | **Type of sample** | **Age**  **at biopsy/**  **autopsy** | **Sex** | **CNS region** | **Disease duration** | **Cancer association** | **Clinical data** | **Imaging abnormalities** | **Treatment** | **Cause of death (if autopsy)** |
| --- | --- | --- | --- | --- | --- | --- | --- | --- | --- | --- | --- | --- |
| AK5_1 | anti-AK5 encephalitis | Positive in serum and CSF | FFPE, biopsy | 58 | F | Temporal | 1 month | No | Headaches, anterograde memory disorders, sleep disorders, and psychiatric disorders. Pleocytosis (120 cells/µl), protein: 103.2 mg/dl- | bilateral, right predominant, mesiotemporal T2/FLAIR hyperintensities with gadolinium enhancement | Steoroids, cyclophosphamide | None (biopsy) |
| Ri_1 | Anti-Ri encephalitis | Positive in serum (Western blot) | FFPE, autopsy | 70 | F | Temporal | < 1 year | Yes (SCLC) | Stuporous (GCS 9), refractory status epilepticus, rapidly progressive dementia. Pleocytosis 10 cells/µl; protein: 50.1 mg/dl | T2 hypersignal in lateral temporal regions predominating on the left. | Steroids | Coma, then cardiorespiratory arrest |
| Ri_2 | anti-Ri encephalitis | Positive in serum (CSF not tested); tissue based assay on fixed rat cerebellum and immunoblot (Ravo); Titer in Serum 1:12800 | FFPE, autopsy | 59 | F | Brainstem | 1.8 years | Yes (breast cancer) | Ataxia, eyelid ptosis and diplopia; locked jaws; progression to orofacial dyskinesias, dysphagia; respiratory depression; CSF: protein elevated, normal cell count; | T2 hyperintense signal alteration in the brainstem (tectum and pons) and cervical spinal cord | Steroids (2 months), followed by IVIG and two doses of rituximab over 3 months. Cyclophosphamide in four separate periods spanning 9 months with no response, leading to discontinuation of treatment. | Unknown (died in a nursing home; needed ventilation) |
| GAD_1 | Anti-GAD encephalitis | Anti-GAD65 Positive in serum and CSF (ELISA) | FFPE, autopsy | 52 | F | Temporal | 4 months | No | Seizures, aphasia, cognitive deficits. Concurrent latent autoimmune diabetes in adults (LADA). Pleocytosis (31 cells/µl, then 100 cells/µl) | T2 hyperintensities and contrast enhancement initially in the right hippocampus, then multifocal (4 months later) | High dose steroids, then IVIg, rituximab | Respiratory failure |
| GAD_2 | Anti-GAD encephalitis | Anti-GAD65 Positive in serum (ELISA) | FFPE, autopsy | 66 | M | Neostriatum | 2 years | Yes (unidentified) | Not available | Not available | Not available | Tumor progression |
| KLHL11_1 | Anti-KLHL11 encephalitis | Positive in serum and CSF | FFPE, autopsy | 33 | M | Hippocampus | 3 years | Yes (seminoma) | Not available | Not available | Not available | Tumor progression |
| KLHL11_2 | Anti-KLHL11 encephalitis | Positive in serum and CSF | FFPE, biopsy | 7 | F | Middle frontal gyrus | < 1 year | No | Pharmacoresistent epilepsy, myoclonus, cognitive deterioration. | Cortical and white matter T2 hyperintensity, then vacuolization in bilateral frontal areas, right hippocampus, right amygdala. Slow progression of diffuse cerebral atrophy on follow up imaging | Steroids, tocilizumab | None (biopsy) |
| **Case ID** | **Disease** | **Antibody titer (if available)** | **Type of sample** | **Age**  **at biopsy/**  **autopsy** | **Sex** | **CNS region** | **Disease duration** | **Cancer association** | **Clinical data** | **Imaging abnormalities** | **Treatment** | **Cause of death (if autopsy)** |
| Ma2_1 | anti-Ma2 encephalitis | Positive in CSF by IHC on tissue, DOTBLOT and CBA, positive in serum by DOTBLOT and CBA | FFPE, autopsy | 73 | M | Temporal (cingular) and hippocampus | 4 months | Yes (ethmoidal cancer) | Limbic encephalitis | Bilateral temporofrontal cortico-subcortical T2 FLAIR hypersignals with cingulate involvement. | Steroids, IVIg, cyclophosphamide, rituximab | Cardiorespiratory arrest due to severe brain damage |
| Ma2_2 | anti-Ma2 encephalitis | Positive in serum and CSF; tissue based assay on fixed rat cerebellum and immunoblot (Ravo); Titer serum 1:204800 | FFPE, autopsy | 77 | F | Brainstem | 1 year | Yes (lung adenocarcinoma) | Brainstem encephalitis: Dysarthria onset one year before death, progressed to hospitalization one month prior with added dysphagia, abducens nerve palsy, and swallowing difficulties. | Hot-cross-bun sign in pons | IVIg | Aspiration pneumonia |
| Hu_1 | Anti-Hu encephalitis | Positive in serum and CSF by IHC on tissue and CBA | FFPE, autopsy | 58 | M | Sensory ganglion | 14 years | Yes (SCLC) | Dysautonomia and sensory neuronopathy, followed by seizures 11 years later. | Not available | Cyclophosphamide, IVIg | Sudden death |
| Hu_2 | anti-Hu encephalitis | Positive in serum and CSF; tissue based assay on fixed rat cerebellum and immunoblot (Ravo)  Titer Serum 1:6400 | FFPE, autopsy | 68 | F | Hippocampus | 7.5 months | Yes (SCLC) | Subacute onset of sensory neuronopathy with hypesthesia, muscle pain, ataxia; progression to tetraplegia; respiratory insufficiency; encephalomyelitis  EEG: diffuse abnormal  CSF: 34 cells/µl; protein: 167 mg/dl | T2 hyperintensity in midbrain and pons | Steroids, PLEX | Pneumonia and cardiac failure |
| CV2_1 | anti-CV2 encephalitis | Positive in serum 1:400, indirect immunofluorescence on primate cerebellum | FFPE, biopsy | 67 | F | Head of caudate nucleus | < 1 year | Yes (SCLC) | Progressive chorea and mnestic disorder | Bilateral hemispheric T2 hyperintense lesions predominantly involving the left head of caudate nucleus and ventral putamen without contrast enhancement | IVIg | None (biopsy) |

Abbreviations: cell-based assay (CBA), cerebrospinal fluid (CSF), Glasgow Coma Scale (GCS), immunohistochemistry (IHC), intravenous immunoglobulin (IVIg), plasma exchange (PLEX), small cell lung cancer (SCLC)
