# Supplementary material for: Neuronal pSTAT1 hallmarks synaptic pathology in autoimmune encephalitis against intracellular antigens
Source: Acta Neuropathol. 2025 Apr 25;149(1):35. doi: 10.1007/s00401-025-02882-7 (PMC12031792; doi:10.1007/s00401-025-02882-7)
Supplement: Supplementary file 2 — Supplementary file2 (DOCX 19 KB) [file 401_2025_2882_MOESM2_ESM.docx]

**Table S2 – Non neurological disease (NND) controls**

| **Control ID** | **Age (years)** | **Sex** | **Brain region** | **Cause of death** | **Matched AE cases** |
| --- | --- | --- | --- | --- | --- |
| NND_1 | 76 | M | Temporal and hippocampus | Brainstem haemorrhage | CASPR2_1  MA2_1  LGI1_1 |
| NND_2 | 33 | M | Temporal and hippocampus | Pneumonia | NMDAR_1  KLHL11_1 |
| NND_3 | 63 | M | Temporal and hippocampus | Heart failure / End stage lung cancer | GABABR_1 |
| NND_4 | 45 | M | Frontal | Exsanguination | AMPAR_1 |
| NND_5 | 69 | F | Temporal and hippocampus | Unknown cause | AMPAR_2 |
| NND_6 | 88 | F | Brainstem (mesencephalon) | Unknown cause | Ma2_2 |
| NND_7 | 71 | F | Hippocampus | Bleeding on cerebellar Arterio-venous malformation | Hu_2 |
| NND_8 | 66 | F | Basal ganglia (Caudate nucleus) | Unknown cause | CV2_1 |
| NND_9 | 58 | F | Temporal and hippocampus | Unknown cause | AK5_1 |
| NND_10 | 70 | F | Temporal and hippocampus | Fall from stairs | Ri_1 |
| NND_11 | 56 | F | Brainstem (Pons) | Unknown cause | Ri_2 |
| NND_12 | 45 | F | Temporal and hippocampus | Unknown cause | GAD_1  NMDAR_3 |
| NND_13 | 67 | M | Basal ganglia (Putamen and caudate) | Cardiorespiratory arrest | GAD_2 |
| NND_14 | 7 | F | Frontal | Unknown cause | KLHL11_2  NMDAR_2 |
