# Supplementary figures and images for: Neuronal pSTAT1 hallmarks synaptic pathology in autoimmune encephalitis against intracellular antigens
Source: Acta Neuropathol. 2025 Apr 25;149(1):35. doi: 10.1007/s00401-025-02882-7 (PMC12031792; doi:10.1007/s00401-025-02882-7)

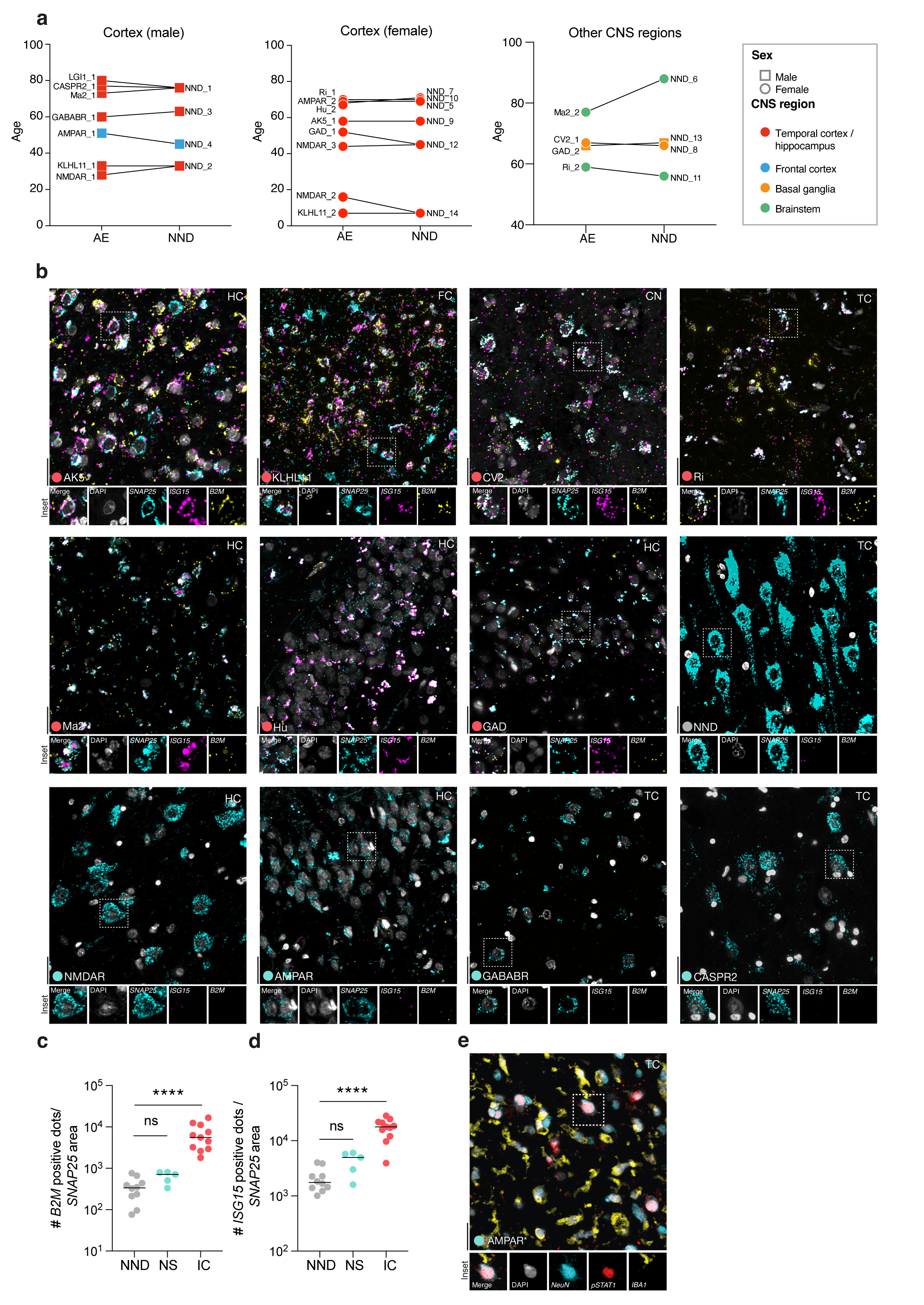

Supplement: Supplementary file 3 — Supplementary file3 (TIF 10372 KB) Figure S1 – Comparative analysis of brain regions and immune markers in autoimmune encephalitis with multiplex fluorescence in situ hybridization and immunostaining. (a) Age-matched AE cases and non-neurological disease (NND) controls in cortical regions such as temporal cortex / hippocampus (red) and frontal cortex (light blue) and non-cortical regions such as basal ganglia (orange) and brainstem (green). Symbols represent individual samples, squares indicate male sex, circles indicate female sex. (b) Representative RNAscope fluorescence in situ hybridization images from NS-AE, IC-AE, and NND brain sections showing co-detection of ISG15 (magenta), B2M (yellow), and the neuronal marker SNAP25 (cyan), with DAPI counterstain (white). (c-–d) Quantification of ISG15 (c) and B2M (d) positive punctae localized within SNAP25-positive neuronal areas. (e) Multiplex immunostaining for pSTAT1(red), neurons (NeuN, cyan), phagocytes (IBA1, yellow) and nuclei (DAPI, white) in tissue sections of fulminant AMPAR-antibody AE (AMPAR*). ***P < 0.001, **P < 0.01, *P < 0.05; ns = not significant by Kruskal-Wallis test with Dunn’s correction for multiple comparisons. Scale bar = 20 µm. Regions: temporal cortex (TC), hippocampus (HC), frontal cortex (FC), caudate nucleus (CN). [file 401_2025_2882_MOESM3_ESM.tif]

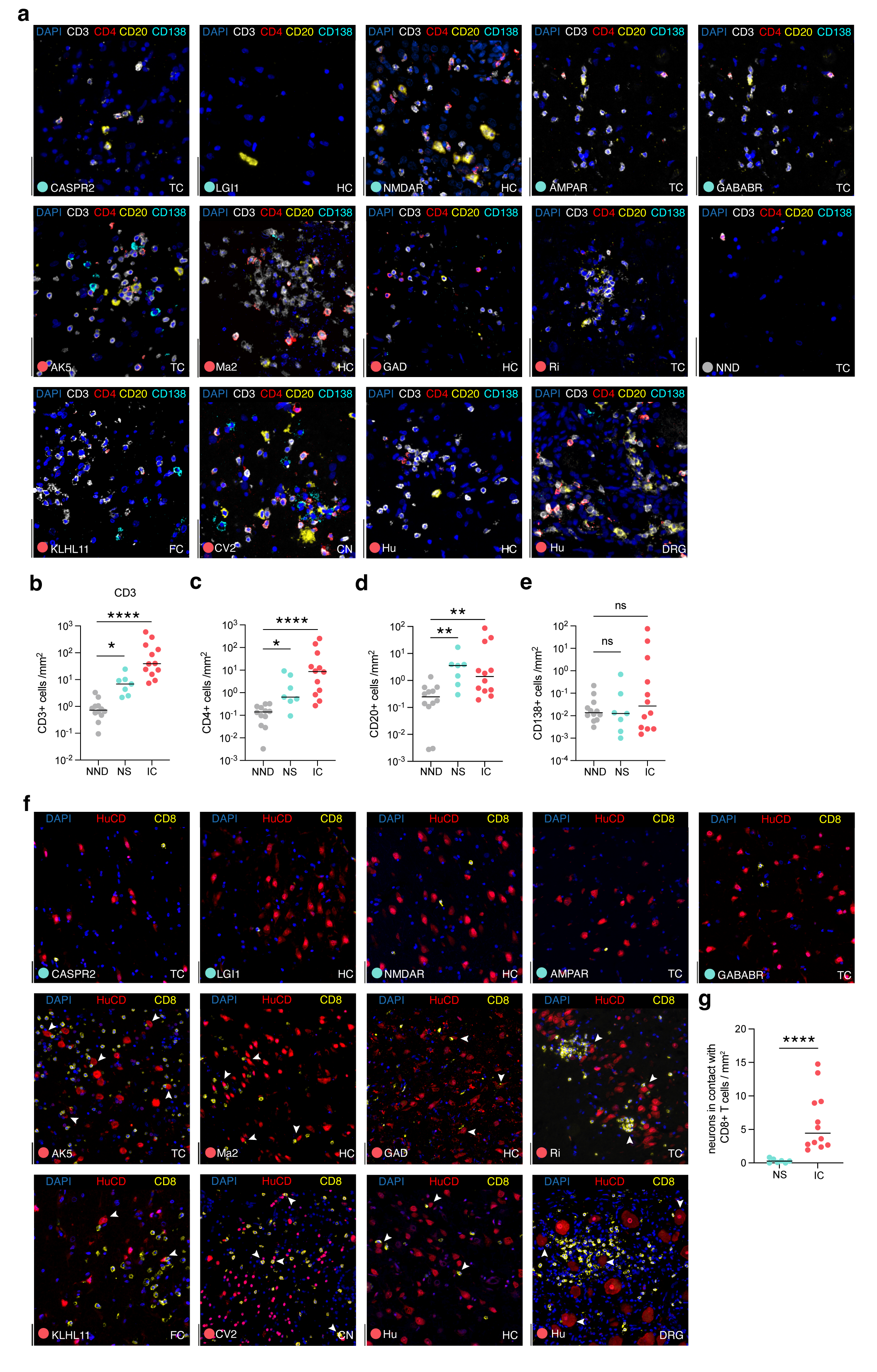

Supplement: Supplementary file 4 — Supplementary file4 (TIF 8755 KB) Figure S2 – Profiling of immune cell infiltrates and contact of CD8+ T cell with neurons in autoimmune encephalitis. (a–d) Representative immunofluorescence images (a) and corresponding quantification (b–d) of brain sections stained for CD3 (white), CD4 (red), CD20 (yellow), CD138 (cyan), and nuclei (DAPI, blue) across the indicated groups. (f–g) Representative images (f) and quantification (g) of sections stained for HuCD (red, neuronal marker), CD8 (yellow), and DAPI (nuclei), highlighting direct contacts between CD8⁺ T cells and neurons. Quantification in (g) shows the density of HuCD⁺ neurons in contact with CD8⁺ T cells. Data are shown on a base 10 logarithmic scale (b-d) or standard scale (g). Lines indicate the median. Symbols represent individual samples. ***P < 0.001, **P < 0.01, *P < 0.05; ns = not significant by Kruskal-Wallis test with Dunn’s correction for multiple comparisons. Scale bar = 50 µm. Regions: temporal cortex (TC), hippocampus (HC), frontal cortex (FC), caudate nucleus (CN), dorsal root ganglion (DRG). [file 401_2025_2882_MOESM4_ESM.tif]

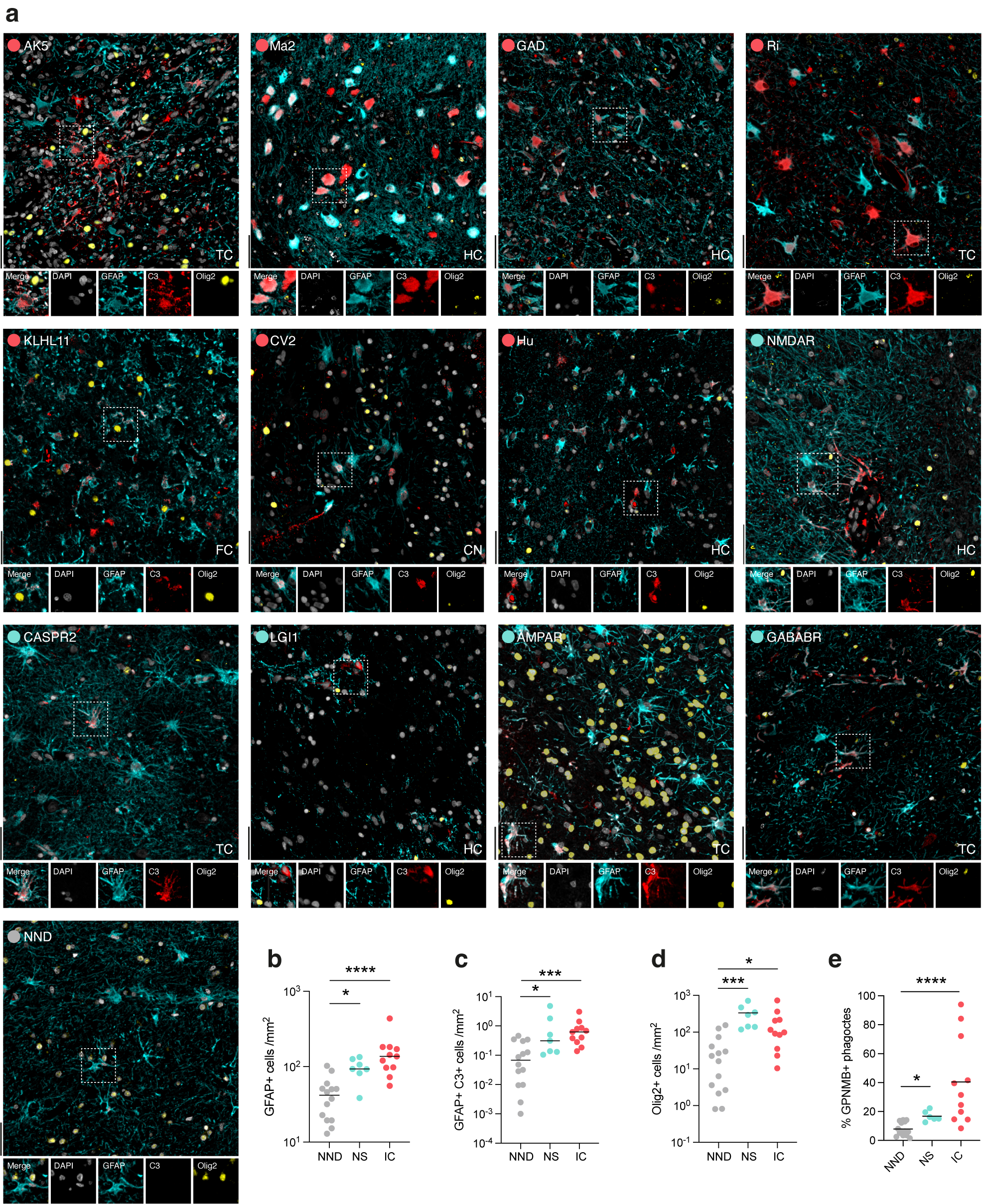

Supplement: Supplementary file 5 — Supplementary file5 (TIF 12383 KB) Figure S3 – Reactive gliosis in autoimmune encephalitis. (a–d) Representative immunofluorescence images (a) and corresponding quantifications (b–e) of brain sections stained for GFAP (cyan), C3 (red), Olig2 (yellow), and nuclei (DAPI, white) across the indicated experimental groups. (c) Neurotoxic astrocytes are identified as GFAP⁺/C3⁺ double-positive cells. (d) Oligodendrocytes are identified by Olig2 positivity. (e) Quantification of brain sections stained for IBA1, GPNMB, and DAPI, highlighting the proportion of phagocytic cells expressing the neurodegenerative marker GPNMB. Data are shown on a base 10 logarithmic scale (b-d) or standard scale (e). Lines indicate the median. Symbols represent individual samples. ***P < 0.001, **P < 0.01, *P < 0.05; ns = not significant by Kruskal-Wallis test with Dunn’s correction for multiple comparisons. Scale bar = 25 µm. Regions: temporal cortex (TC), hippocampus (HC), frontal cortex (FC), caudate nucleus (CN). [file 401_2025_2882_MOESM5_ESM.tif]

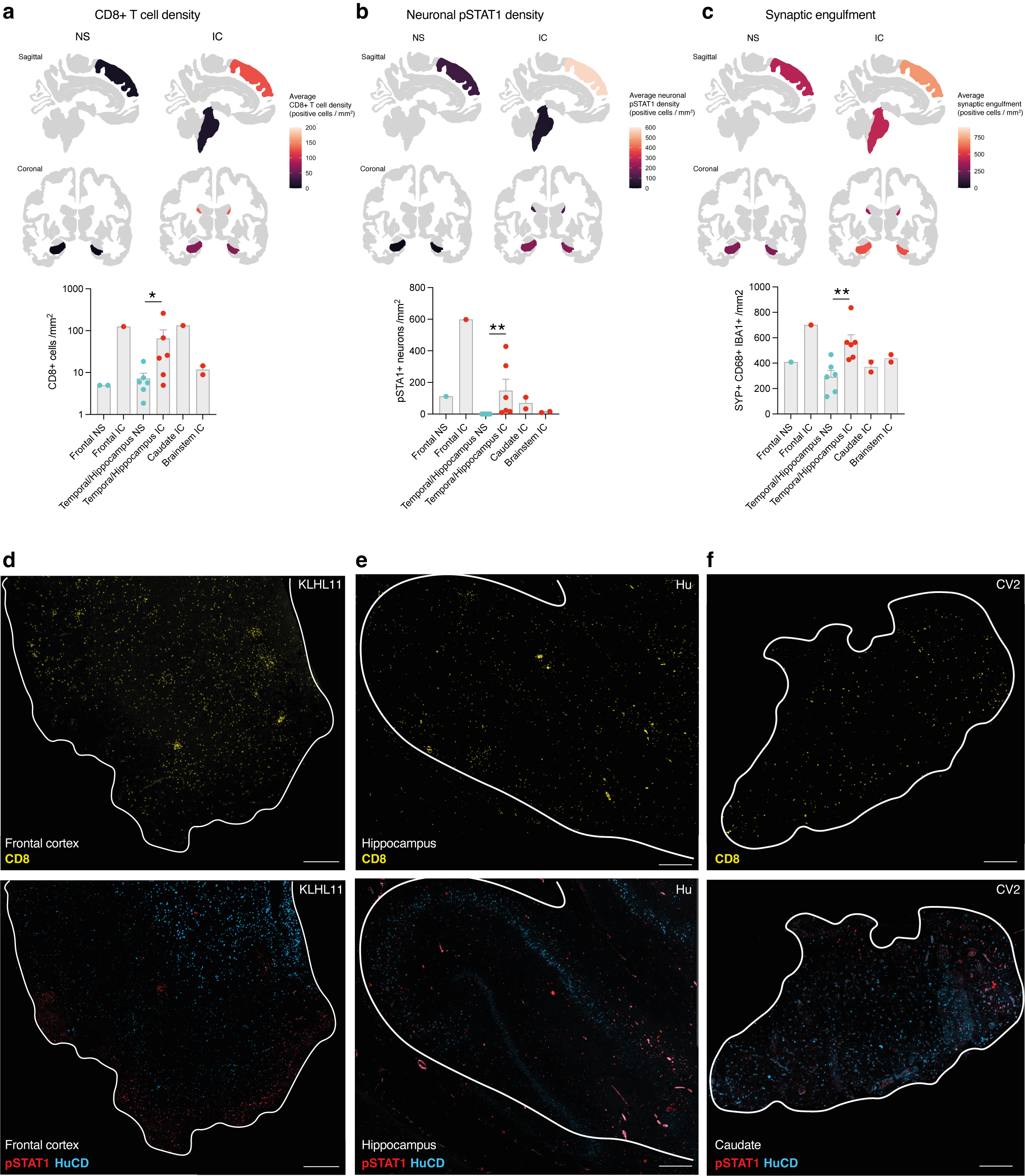

Supplement: Supplementary file 6 — Supplementary file6 (TIF 6745 KB) Figure S4 - Regional distribution of CD8⁺ T cell infiltration, neuronal pSTAT1 activation, and synaptic pathology. (a–c) Quantification of CD8⁺ T cell density (a), neuronal pSTAT1⁺ cells (b), and synaptic engulfment by phagocytes (IBA1⁺SYP⁺CD68⁺) (c) in the frontal cortex, hippocampus/temporal cortex, caudate, and brainstem of NS and IC groups. The color scale denotes the severity of histopathological alterations across regions. (d-f) Representative low-magnification immunofluorescence images illustrating the distribution of CD8⁺ T cells and neuronal pSTAT1 expression in brain sections of frontal cortex (d), hippocampus (c) and caudate nucleus (e) stained for CD8 (yellow), pSTAT1 (red), and HuC/D (light blue, neuronal marker). Scale bar = 500 µm. Data are shown on a base 10 logarithmic scale (a-c). Lines indicate the median. Symbols represent individual samples. **P < 0.01, *P < 0.05; comparisons between NS and IC in the hippocampus and temporal cortex were performed using the Mann–Whitney U non-parametric test. [file 401_2025_2882_MOESM6_ESM.tif]

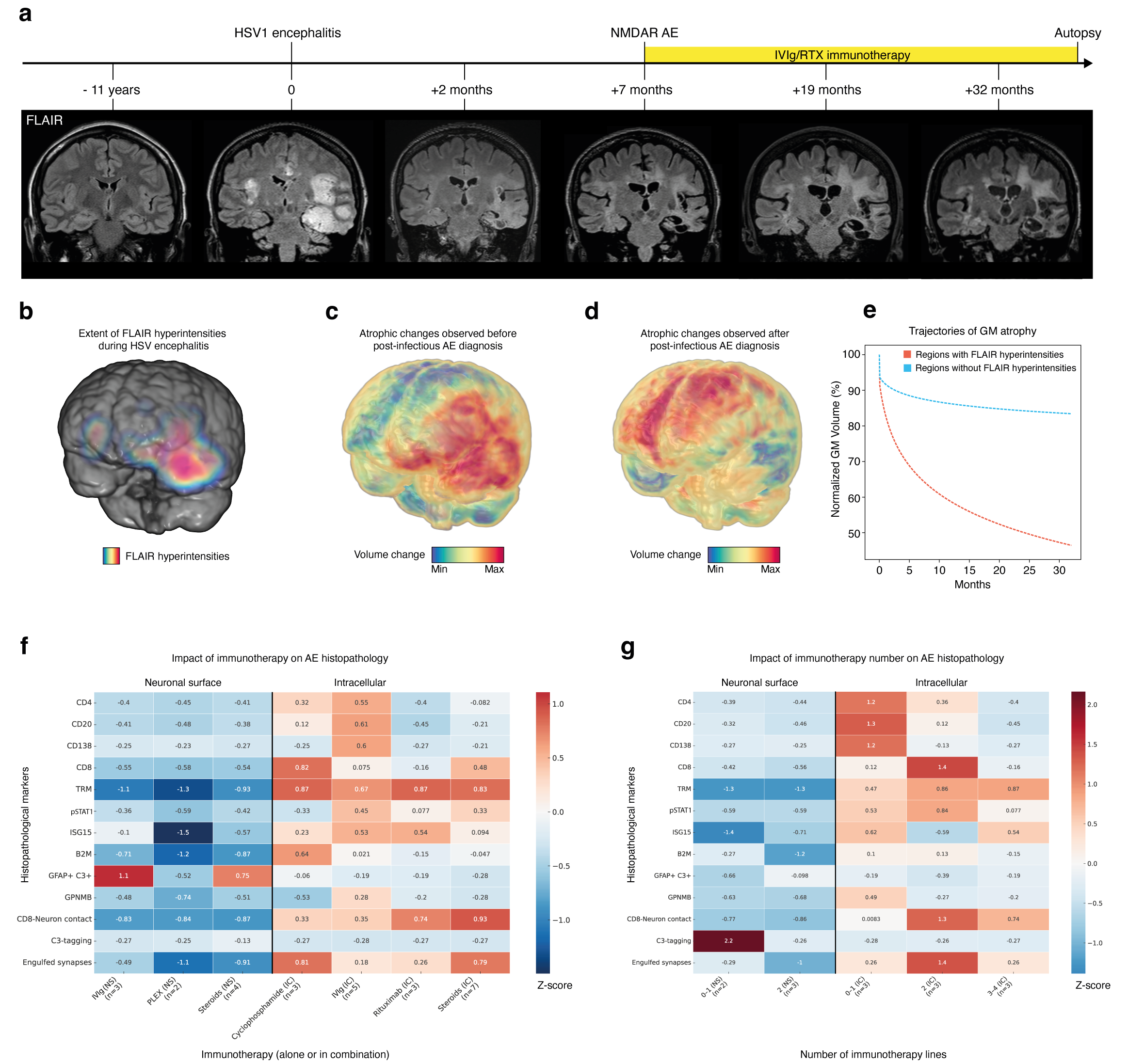

Supplement: Supplementary file 7 — Supplementary file7 (TIF 4996 KB) Figure S5 – Neurodegenerative sequelae in post-infectious autoimmune encephalitis and impact of immunotherapies on histopathological markers. (a) Disease Progression Timeline: MRI scans depicting disease progression in a patient with post-infectious autoimmune encephalitis, including initial baseline (-11 years), HSV1 encephalitis diagnosis, follow-up (+2 months), subsequent post-infectious (PI) NMDAR-antibody encephalitis diagnosis (+7 months), and long-term follow-up (+19 and 32 months) culminating in an autopsy 36 months after the diagnosis of HSV1 encephalitis. (b) 3D volume rendering of FLAIR hyperintensities during HSV1 encephalitis overlayed on the brain template. (c) Atrophic changes during the interval between the diagnosis of HSV1 encephalitis and post-infectious AE are depicted on the brain template. (d) Atrophic changes after the diagnosis of post-infectious AE are depicted on the brain template. (e) Progressive grey matter (GM) volume changes in regions initially affected and non-affected by FLAIR hyperintensities at the time of HSV1 encephalitis diagnosis are logarithmically curve-fitted to predict their evolution over time. (f–g) Heatmaps display the effect of immunotherapy (f) and number of immunotherapy lines (g) on histopathological markers in neuronal surface (NS) and intracellular (IC) autoimmune encephalitis (AE), presented as Z-scores. (f) Impact of specific immunotherapies (administered alone or in combination) on histopathological markers, stratified by NS and IC subtypes. Immunotherapies include intravenous immunoglobulins (IVIg), plasmapheresis (PLEX), corticosteroids, cyclophosphamide, and rituximab. (g) Association between the number of immunotherapy lines (0–1, 2, or ≥3) and histopathological marker expression in NS and IC AE subtypes. Histopathological markers include T and B cell markers (CD4, CD8, CD20, CD138), tissue-resident memory CD8⁺ T cells (TRM), interferon signaling (pSTAT1, ISG15, B2M), marke [file 401_2025_2882_MOESM7_ESM.tif]
